# Equitable hospital length of stay prediction for patients with learning disabilities and multiple long-term conditions using machine learning

**DOI:** 10.3389/fdgth.2025.1538793

**Published:** 2025-02-14

**Authors:** Emeka Abakasanga, Rania Kousovista, Georgina Cosma, Ashley Akbari, Francesco Zaccardi, Navjot Kaur, Danielle Fitt, Gyuchan Thomas Jun, Reza Kiani, Satheesh Gangadharan

**Affiliations:** ^1^ Computer Science Department, School of Science, Loughborough University, Loughborough, United Kingdom; ^2^ Faculty of Medicine, Health and Life Science, Swansea University, Swansea, United Kingdom; ^3^ Leicester Real World Evidence Unit, Diabetes Research Centre, University of Leicester, Leicester, United Kingdom; ^4^ School of Design and Creative Arts, Loughborough University, Loughborough, United Kingdom; ^5^ Learning Disability Service (Agnes Unit), Leicestershire Partnership NHS Trust, Leicester, United Kingdom

**Keywords:** learning disabilities, length of stay, bias mitigation, threshold optimiser, exponentiated gradient

## Abstract

**Purpose:**

Individuals with learning disabilities (LD) often face higher rates of premature mortality and prolonged hospital stays compared to the general population. Predicting the length of stay (LOS) for patients with LD and multiple long-term conditions (MLTCs) is critical for improving patient care and optimising medical resource allocation. However, there is limited research on the application of machine learning (ML) models to this population. Furthermore, approaches designed for the general population often lack generalisability and fairness, particularly when applied across sensitive groups within their cohort.

**Method:**

This study analyses hospitalisations of 9,618 patients with LD in Wales using electronic health records (EHR) from the SAIL Databank. A Random Forest (RF) ML model was developed to predict hospital LOS, incorporating demographics, medication history, lifestyle factors, and 39 long-term conditions. To address fairness concerns, two bias mitigation techniques were applied: a post-processing threshold optimiser and an in-processing reductions method using an exponentiated gradient. These methods aimed to minimise performance discrepancies across ethnic groups while ensuring robust model performance.

**Results:**

The RF model outperformed other state-of-the-art models, achieving an area under the curve of 0.759 for males and 0.756 for females, a false negative rate of 0.224 for males and 0.229 for females, and a balanced accuracy of 0.690 for males and 0.689 for females. Bias mitigation algorithms reduced disparities in prediction performance across ethnic groups, with the threshold optimiser yielding the most notable improvements. Performance metrics, including false positive rate and balanced accuracy, showed significant enhancements in fairness for the male cohort.

**Conclusion:**

This study demonstrates the feasibility of applying ML models to predict LOS for patients with LD and MLTCs, while addressing fairness through bias mitigation techniques. The findings highlight the potential for equitable healthcare predictions using EHR data, paving the way for improved clinical decision-making and resource management.

## Introduction

Learning disability (LD), also referred to as intellectual disabilities in some contexts, have been defined by the Learning Disabilities Observatory (1) as the presence of: “a significantly reduced ability to understand new or complex information, to learn new skills (impaired intelligence), with a reduced ability to cope independently (impaired social functioning); which started before adulthood, with a lasting effect on development.” There are approximately 1.1 million adults aged 18 years and older living with an LD in the UK, including over 54,000 individuals from Wales (2). Existing sources show that individuals with LD often experience poorer physical and mental health, as well as higher rates of multiple long-term conditions (MLTCs) and avoidable mortality compared to those without LD (3–10). This demographic presents unique needs and challenges that impact their hospitalisations (11).

Effectively managing healthcare resources while ensuring optimal patient outcomes poses particular challenges for patients with LD. An important outcome of interest for patients with LD is a reliable prediction of the length of hospital stay (LOS) of their admission and the underlying factors that could influence their LOS (12). Predicting the LOS can lead to enhanced healthcare services and further initiate proactive measures to prevent prolonged LOS. A recent study conducted in the UK discovered that at any given time, approximately 2,000 patients with LD and/or autism in long-stay hospitals have been hospitalised, with over half having spent over 2 years in hospital care. This includes 350 LD patients who have been in long-stay hospitals for more than a decade (13). The extended LOS in their study was attributed to either the patient’s personal characteristics or limitations of the system supporting them. Another study found that other general factors contributing to prolonged hospital stay for patients who are medically fit for discharge included hospital-acquired infections, falls, and other medical errors (14). Conversely, there is also a downside to patients being discharged prematurely, as it may result in readmissions or, in severe cases, preventable deaths (15, 16). Although these studies were carried out on the general population, the conclusion and outcome may still apply to people with LD with MLTCs.

The above-mentioned studies highlight the importance of proactively managing patient discharges as early as possible during their hospitalisation to optimise the LOS.

Several studies have utilised machine learning (ML) models to predict the LOS of individual patients in the general population by analysing large datasets of hospital admission records (17–19). These models typically assess the influence of various factors—such as sex, age, diagnosis, admission method, and illness severity—on the likelihood of hospital stays exceeding a predefined threshold. However, there is limited research focusing on the prediction of hospitalisations for adults with learning disabilities using ML. This cohort faces unique challenges, underscoring the importance of tracking their clinical pathways to better inform medical interventions and reduce average hospital durations.

Most existing LOS prediction studies using ML focus on specific long-term conditions, medical units, or single hospitals or geographic regions. As a result, many of these models may not be applicable to patients with varied MLTCs [e.g., those with multiple co-occurring long-term conditions (LTCs)] or to individuals from diverse socio-geographic backgrounds. Furthermore, a vast majority of these studies fail to provide adequate explanations regarding the accuracy of ML models across distinct patient subgroups, such as ethnicity, age, and sex (20–22). This lack of transparency raises concerns about the applicability of these models to sensitive groups. Such concerns are particularly important from an ethical standpoint, as the models may be biased towards underrepresented communities. These limitations underscore the urgent need for fairness in healthcare ML models, ensuring that predictions are explainable, and that model performance is thoroughly evaluated and optimised across diverse patient groups (23).

This study aims to predict the LOS of patients with LD and MLTCs in Wales using ML models. The model is built on a dataset of 62,243 hospital admission records from 9,618 patients with LD. It incorporates 39 long-term conditions (S1), identified through a literature review and expert consensus (24, 25), as well as demographics, medication history, prior hospitalisations, and lifestyle factors to account for potential confounding variables.

Bias in ML classification models in healthcare often stems from imbalanced datasets, systemic inequities, or suboptimal feature selection, which can result in unfair outcomes for sensitive patient groups (e.g., ethnic minorities, sex, socioeconomic status, or specific medical conditions). To address these issues, bias mitigation techniques are typically applied at three stages of the ML pipeline: preprocessing (before model training), in-processing (during training), and post-processing (after training) (26, 27). This study focuses on fairness in predicting outcomes across sensitive ethnic groups by employing two bias mitigation techniques. The first is the threshold optimiser, a post-processing method applied after model training. It adjusts the classifier’s output scores based on a fairness constraint to reduce bias (27, 28). The second is the reductions method with exponentiated gradient (EG), an in-processing approach that modifies the classifier’s weights during training to ensure fairness in real time (22, 27). Both techniques were rigorously evaluated to assess their impact on model performance across different ethnic groups.

The structured electronic healthcare datasets used in this study presented inherent complexities due to various inconsistencies in patient records over time. These inconsistencies can stem from factors such as data entry errors, biased data collection, missing documentation, issues with patient compliance, and changes in patient status that are not captured in the electronic health record (EHR) (29). To address these complexities, we employed statistical preprocessing techniques, systematically handled missing data, and applied algorithms to mitigate biases. The resulting ML model demonstrated strong performance, validating our approach to managing complex healthcare data. The contributions of this paper are as follows:

• Analysis of hospitalised Welsh LD patient population—provided statistics on demographics, prevalence of 39 LTCs, previous hospitalisation data including prior admissions, episodes, days, and condition prevalence.
• Identification of primary conditions treated during hospitalisation and the prevalent LTCs for hospitalised patients with LD, along with admission rates per patient.
• Identification of prevalent LTCs linked to prolonged hospital stays (≥129 days).
• Statistical analysis to identify factors associated with hospital stays ≥4 days using non-parametric tests.
• Development and evaluation of machine learning models to predict whether a patient’s LOS would be <4 days or ≥4 days, using patient data available up to the first 24 h of admission.
• Assessment of model performance differences across ethnic groups. Application and comparison of two bias mitigation algorithms: threshold optimisation and reductions algorithm using an exponentiated gradient.
• Demonstrated potential of applying ML with effective bias mitigation on electronic health records data to promote equitable prediction across groups when predicting LOS.

## Methods

The LOS is defined as the number of days an inpatient is hospitalised during a single admission (12). For each admission, the LOS value was obtained by calculating the difference between the admission and discharge dates. An LOS threshold ψ was obtained by taking the ceiling value of the mean LOS across all extracted admission records, excluding the “outliers.” With the mean LOS of 3.015 days (standard deviation of 4.064 days), ψ was set to ψ=⌈3.015⌉=4 days. Therefore, the LOS threshold (ψ) was set to 4 days. Throughout this study, a “long stay” refers to hospitalisation with LOS ≥4 days, while a “short stay” denotes LOS <4 days. The “long-stay rate,” is defined as the percentage of admissions lasting at least 4 days, calculated using

(1) $$\text{Long-stay rate}=\frac{\text{Number of admissions with LOS}\geq 4}{\text{Total number of admissions}}$$

**Figure 1:**
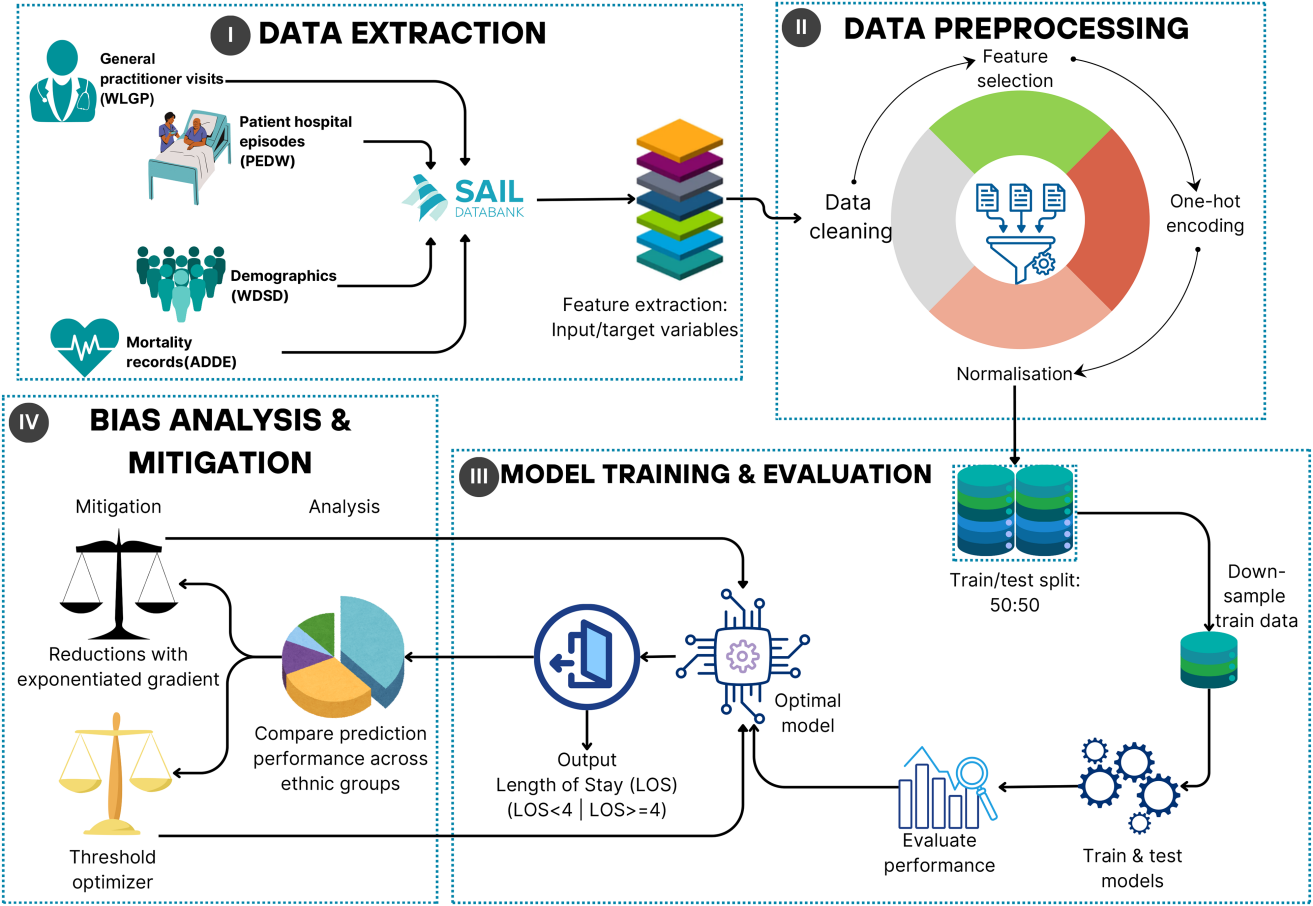
Machine learning framework diagram.

This study addresses three key research questions: (1) How can ML models be used to accurately predict the LOS for patients with LD and MLTCs using EHR data? (2) What are the key sources of bias in ML models for LOS prediction, and how do they impact accuracy and fairness across sensitive groups, such as ethnic minorities? (3) Can bias mitigation techniques effectively improve fairness across ethnic groups while maintaining or enhancing predictive performance? To tackle these challenges, a structured methodology was developed as illustrated in Figure 1, integrating data extraction, preprocessing, ML model development, and applying bias mitigation techniques to ensure robust and equitable predictions for this population.

### Stage I: data extraction

#### SAIL database

This study utilised EHR data sources of hospital admissions for patients with LD, contained in the Secure Anonymised Information Linkage (SAIL) Databank, the national Trusted Research Environment (TRE) for Wales, enabling the use of anonymised individual-level, population-scale, and linked data sources (30–32). SAIL partners with the National Health Service (NHS) and Welsh government to organise routinely collected longitudinal health and administrative data for approximately 5 million Welsh residents, accessed securely under strict conditions compliant with the General Data Protection Regulation (GDPR). Specifically for this study the Welsh Longitudinal General Practice (WLGP) data containing information on primary care General Practice (GP) records, the Patient Episode Database for Wales (PEDW) containing information on secondary care inpatient hospitalisation admissions, the Welsh Demographic Service Dataset (WDSD) containing patient demographic, residency, and registration history, and the Annual District Death Extract (ADDE) containing mortality records from the Office for National Statistics (ONS) were accessed. An Anonymised Linking Field (ALF) facilitates longitudinal anonymised linkage through all data sources in SAIL (33). Data captured within primary care is via read codes. The International Classification of Diseases version 10 (ICD-10) codes capture diagnosis, and Office of Population Censuses and Surveys (OPCS) Classification of Interventions and Procedures version 4 (OPCS-4) captures operations in hospital admissions.

#### Inclusion and exclusion criteria

**Figure 2:**
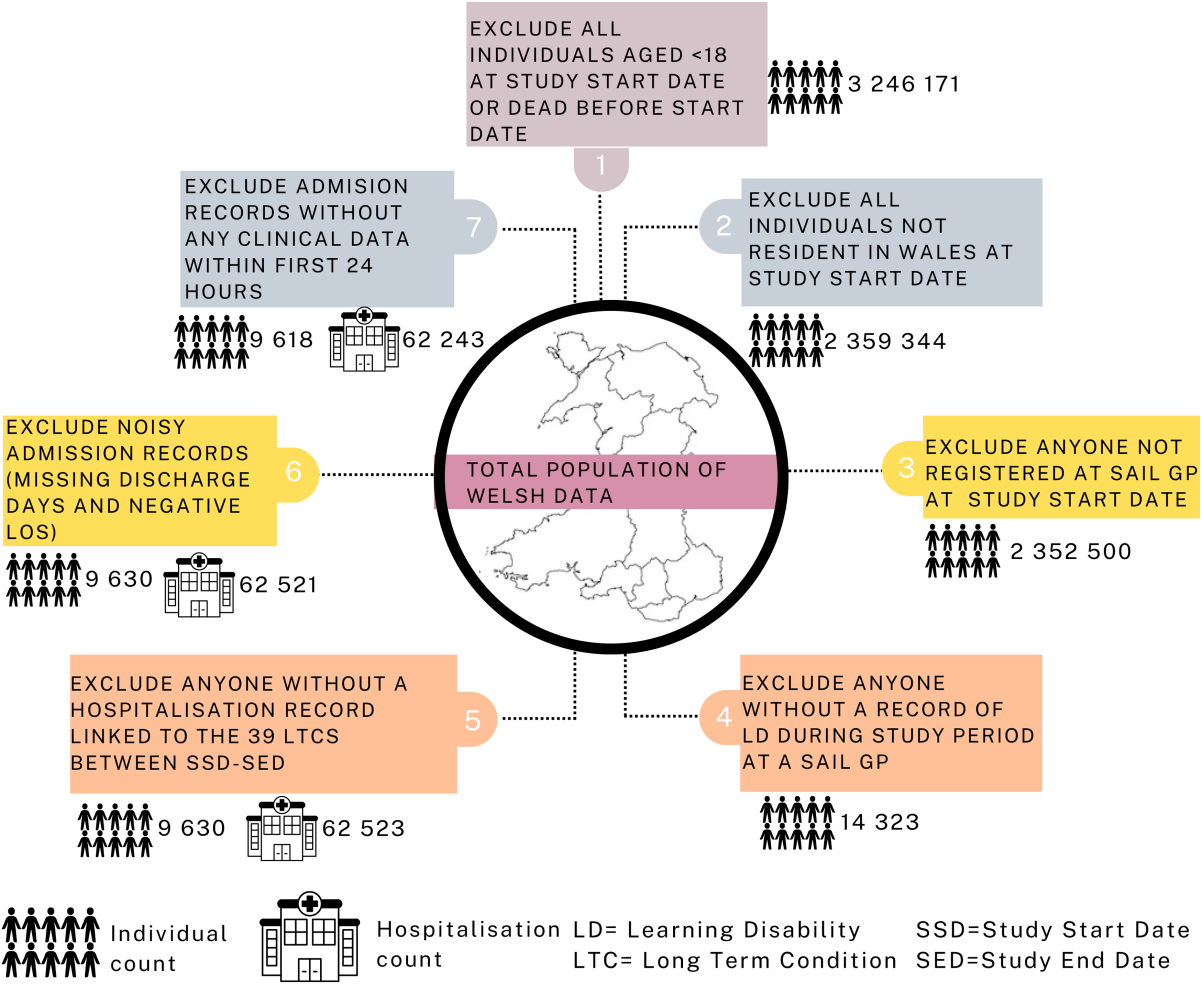
Consort flow diagram.

The study focused on Welsh residents aged 18 years or older identified with LD during the period from 1 January 2000 to 31 December 2021, as depicted in Figure 2: Exclusions were implemented for individuals younger than 18 years, those not residing in Wales, individuals not registered with a SAIL GP at the study start date, and those without LD. This process led to a total of 14,323 unique patients during the data extraction phase. Subsequently, patients without any hospital admission records between the study start and end dates were omitted. The resulting admissions data were then associated with 39 LTCs (Supplementary Table S1), documented primarily through Read codes in primary care and ICD-10 codes in secondary care (34, 35). The dataset comprised 62,523 unique admissions from 9,630 LD patients, spanning the period from January 2000 to December 2021. Exclusions were applied to records with missing discharge dates and negative LOS, resulting in a cohort of 62,521 unique admissions from 9,630 LD patients. Finally, admissions with no data within the first 24 h were excluded, yielding the final cohort of 9,618 unique patients (4,929 males, 4,689 females) with 62,243 unique admissions (32,275 males, 29,968 females). Refer to Supplementary Table S2 for further details on demographic distribution. Additionally, Supplementary Table S3 provides a comprehensive list of all variables extracted for each patient across their admissions.

#### Dataset demographic description

**Age group**. Age was categorised into seven groups, as shown in Supplementary Table S2. Patient and admission counts generally followed a normal distribution across age groups for both sexes, with fewer admissions in ages above 60–69 and below 30. Possible factors for lower admission rates in ages above 60–69 include home care reducing hospital need and mortality (see Supplementary Table S4 for mortality statistics on this study cohort). The statistics on mortality obtained in this study are consistent with the life expectancy statistics (66 and 67 years for LD males and females, respectively) (36). Notably, ages above 60–69 exhibited higher rates of long stays, similar for both sexes.

**Welsh Index of Multiple Deprivation**. Patients’ socioeconomic status was described using the Welsh Index of Multiple Deprivation (WIMD) version 2019 for Wales (37), an area-level weighted index across seven deprivation domains assigned based on the individual’s residence using their Lower-layer Super Output Areas (LSOA) version 2011, with each LSOA representing an area of around 1,500 people. The seven deprivation domains include income, employment, health and disability, education skills and training, barriers to housing and services, living environment, and crime. WIMD quintiles were used, categorising the area of residence from 1 (most deprived) to 5 (least deprived) as shown in Supplementary Table S2. Patients without an LSOA or associated WIMD quintile were grouped as “Unknown.” The highest representation of hospital admissions was from the most deprived quintile (quintile 1), comprising ∼28% of admissions for males and ∼27% of admissions for females, indicating heightened hospital demand with increasing deprivation. This aligns with research done on the general population, attributing higher admission rates in impoverished areas to factors such as inadequate allied healthcare and local resources and potential underuse of community medical resources (38, 39). Supplementary Table S4 also shows higher mortality rates with increasing deprivation for the study cohort. This finding is consistent with a recently published study on the impact of deprivation on mortality (7).

**Ethnic group**. Ethnic groups were classified using the ONS, UK categorisation. The methodology by Akbari et al. (40) was used to extract and harmonise the ethnic group details from the various data sources available to the project. The cohort was not uniformly represented (i.e., unbalanced) in terms of ethnic groups: ∼73% of patients (∼79% of admissions) and ∼74% of patients (∼80% of admissions) were from the “White” group for the male and the female sex, respectively (see Supplementary Table S2). Within the cohort, ∼25% of males and ∼24% of females had no ethnic group records, classified as “Unknown” (19.24% and 17.53% of male and female admissions, respectively). The remaining ethnic groups (Black, Asian, Mixed, Other) each represented <3% of patients and admissions for both sexes. Long-stay rates varied widely across the ethnic groups, with the “Black” group having the highest rate (M: 52% and F: 65.9%; M denotes male sex and F denotes female sex) and the “Other” group the lowest (M: 34.2% and F: 30.4%). A similar finding was observed in a study on inpatient discharges for the general patient population in the United States, which revealed that Black patients had significantly longer LOSs compared to other groups (41).

#### Feature extraction

**Extracting the inputs**. The ML models in this study utilise a dataset of 9,618 patients with LD (see Supplementary Table S2) and a total of 62,243 hospital admission records to predict each patient’s LOS (i.e., target variable). For each admission, data relating to the patient’s health up until the first 24 h of admission were extracted to be applied as inputs to the ML models. As detailed in Supplementary Table S3, this data includes variables describing: the patient’s lifestyle and history [body mass index (BMI), smoking, alcohol consumption, physical exercise, autism]; prior 1-year and 3-year hospitalisation data (previous admissions and hospital episodes, cumulative hospital days from past admissions, and the number of MLTCs from previous admissions); prescribed antipsychotic, antidepressant, and anti-manic/anti-epileptic medications (Supplementary Table S5 provides the medications list); and other variables from first 24 h of current admission, indicating the prevalence of the 39 LTCs (see Supplementary Table S1 for the full list of LTCs). Age group was also included as an input into the model (i.e., as a predictor) because MLTC counts increase with age (5, 42) and consequently have an impact on the length of stay. There is a deficit of studies that apply ML to predict the LOS for adults with LD. Therefore, the variables used as inputs in this study were selected through a review by a professional advisory board, supplemented by insights from existing studies utilising ML for hospital predictions in the general population (18, 19, 43, 44).

**Extracting the target variable**. The LOS variable indicating the number of hospital days was replaced with a binary target variable (LOSClass) for machine learning classification purposes, as follows: values with an LOS ≥ 4 days where replaced with 1 indicating long stay, and values with LOS < 4 days where replaced with 0 indicating short stay, as shown in Supplementary Table S3.

### Stage II: preprocessing the inputs for ML

#### Step 1: data cleaning

Supplementary Table S3 describes the ML model input and target variables, and how they were preprocessed for ML training and analysis. Supplementary Table S3 also shows the demographic variables and these were not applied as inputs to the ML model (except age group) but were utilised for describing the cohort and for bias analysis of ML model performance. To clean the data two steps were carried out: coding the longitudinal variables and handling missing data

**Preprocessing longitudinal risk factors**. BMI, alcohol consumption, smoking, physical activity, and medications are longitudinal variables, gathered and coded by GPs or practice nurses. These risk factors change over time across patients. Hence, the variable “BMI” was coded categorically using the patient’s BMI value documented closest to the admission date. This enables the model to use the most recent patient BMI description for each unique admission. Alcohol and smoking records, which are self-reported risk factors, were found to be noisy and inconsistent across patients. For example, some chronological records indicated declarations such as “ex-smoker,” followed by “smoker,” and then “never smoked,” suggesting potential dishonesty or imputation errors. To address this, these risk factors were recoded based on historical data rather than current status, assuming that past behaviours could have a lasting impact on the patient’s physiology (45, 46). The variables “ALCOHOL_HISTORY” and “SMOKING_HISTORY” were categorised based on intake history up to the admission date (see Supplementary Figures S1, S2). “PHYSICAL” was coded categorically using the patient’s physical activity status (i.e., if the patient engages in light or regular exercise) documented nearest admission. The “MEDICATIONS” variable was coded as binary for intake history at each admission and assumed lifetime use from the first prescription.

**Missing values**. Missing values for all categorical variables were classified into “unknown” categories for their respective variables This allows models to utilise the partial information from observations with missing data rather than discarding or imputing them during preprocessing, consequently introducing more bias into the model. There were no missing data for the numerical variables.

#### Step 2: feature selection

A correlation test explored relationships between the numerical features (columns) in the dataset. Correlation analysis aids ML model building by detecting redundant inputs, simplifying interpretations, and improving target prediction performance. Supplementary Table S3 describes all features for predicting LOS. The Kolmogorov–Smirnov test assessed feature normality before selecting an appropriate correlation analysis. As shown in Supplementary Table S6, all features had non-normal distributions. Consequently, the non-parametric Spearman rank correlation coefficient was evaluated. Supplementary Figures S3, S4 illustrate no statistically significant associations between any input and the outcome (*LOSClass*) for male and female cohorts. However, several input variable pairs exhibited collinearity, with correlation coefficients exceeding ±0.5 for both groups. This study found that the interaction of the highly correlated variable pairs provided useful information to the model for the cohort examined. “While their correlation did not imply causation, this observation, combined with feedback from clinicians on the professional advisory panel, supported their inclusion in the analysis.”

#### Step 3: convert categorical variables to one-hot encoded variables

ML models require numerical inputs/target variables. To enable this, the categorical input variables were converted into numeric representations via one-hot encoding. The steps for one-hot encoding a categorical variable using the variable “PHYSICAL” as an example, are as follows:

1. Identify unique categories and count per variable, e.g., “PHYSICAL” has three categories: Yes, No, Unknown (Supplementary Table S3).
2. Create a new binary variable for each category. For each admission, only one of the new binary variables is “hot” (1), indicating its category. This is illustrated by example in Supplementary Tables S7, S8 for the variable “PHYSICAL” recorded over three admissions.
3. Concatenate these new binary columns to the dataset. Hence, from the example, instead of one three-value variable, “PHYSICAL” is now encoded into three separate binaries that ML models can easily use.

#### Step 4: data normalisation

Z-score normalisation was applied to all numerical input variables in Supplementary Table S3. Through this, each numerical variable was centred to have zero mean and unit variance. The standardised data retains the skewness and kurtosis shape properties of the original dataset. Data normalisation ensures that variables with different scales are rescaled to a common range, preventing larger features from dominating ML models. This improves model performance, training stability, and convergence while reducing the impact of outliers. In this study, normalisation ensured equal contributions from all features, enhancing accuracy, fairness, and generalisability in predicting the LOS.

### Stage III: model training and evaluation methodology

#### Step 1: initial train and test split

The preprocessed dataset comprising the model inputs was split into two separate sets: a training set and a test set, utilising a stratified 50–50 partition. Hence, the training and test set each had 16,121 samples for the male dataset and 15,094 samples for the female group.

#### Step 2: downsampling of the training set

To ensure a balanced representation across the two classes of the target variable (long stay: LOSClass = 1 and short stay: LOSClass = 0), the majority class (short stay) in the training set was downsampled. Specifically, the number of training samples belonging to the short-stay class was reduced to match the number of examples from the smaller, long-stay class. Applying this balanced downsampling serves to mitigate potential modelling inefficiencies caused by class imbalance, where models may ignore or not properly learn underrepresented classes. Also, by harmonising class distributions, downsampling can improve model evaluation metrics related to average performance across classes, such as balanced accuracy, as both classes are weighted and assessed equally. Supplementary Tables S9, S10 show the demographic distribution of the training and test data applied in classifying the LOS for males and females, respectively.

#### Step 3: model training and testing

To identify an optimal ML model for predicting hospitalisation duration (LOSClass), eight classifiers were evaluated including logistic regression (LR) (47, 48), support vector machine (SVM) (47, 49), Random Forest (RF) (47, 49), eXtreme Gradient Boosting (XGBoost) (47), histogram-based gradient boosting (HISTGBoost) (48), XGBoost (48), k-nearest neighbor (KNN) (47), and a sequential neural network (NN) model (49). Supplementary Table S11 indicates the parameter configurations set for each classifier. Supplementary Tables S12, S13 detail the parameter configurations for the NN.

#### Step 4: performance evaluation

Several evaluation metrics were employed to evaluate each classifier’s performance on the test set. Let |TP| denote the number of unique admissions for which the long stay (i.e., LOSClass = 1) was correctly classified; |TN| be the number of short stays (LOSClass = 0) correctly classified; |FP| be the number of short stays incorrectly classified as long stays; |FN| be the number of long stays incorrectly classified as short stays; |P| be the total number of long-stay admissions, where |P|=|TP|+|FN|; and |N| represents the total number of short-stay admissions, where |N|=|TN|+|FP|. The following metrics defined in Equations 2–6 were utilised to evaluate the performance of the ML models.

(2) $$\text{True positive rate ( TPR) }=\frac{\left|TP\right|}{\left|TP|+|FN\right|}\in (0,1)$$

(3) $$\text{True negative rate ( TNR) }=\frac{\left|TN\right|}{\left|TN|+|FP\right|}\in (0,1)$$

(4) $$\text{Balanced accuracy}=\frac{\text{TPR + TNR}}{2}\in (0,1).$$

The closer the values of the abovementioned metrics are to 1, the better the performance of the model. The FNR and FPR are given by the expression

(5) $$\text{FNR}=1-\text{TPR}=\frac{\left|FN\right|}{\left|TP|+|FN\right|},\in (0,1),$$

(6) $$\text{FPR}=1-\text{TNR}=\frac{\left|FP\right|}{\left|TN|+|FP\right|},\in (0,1).$$

The closer the values of the FNR and the FPR are to 0, the better the performance of the model. Another important evaluative measure is the receiver operating characteristic (ROC) curve, which plots the true positive rate (TPR) against the FPR at different threshold values. This creates a curve from (0,0) to (1,1). The area under the ROC curve (AUC) measures the two-dimensional area beneath this curve. A greater AUC shows better performance in predicting the long (LOS ≥4 days) and short (LOS < 4 days) hospital stays.

**Assessing model generisability**. After the optimal model (RF) was selected by comparing the metrics, it was further evaluated using repeated random train/test splits to scrutinise the model’s generalisability. Specifically, the dataset was randomly split into dedicated 50% sized training sets and 50% sized testing sets a total of 10 times. This generated 10 distinct train/test set combinations, allowing for evaluation across different data partitions. For each of the 10 train/test splits, the corresponding training set was downsampled as described in step 2 of Stage III and used to train the model. The model performance was evaluated at each iteration using its corresponding test set. Finally, the evaluation performance across the 10 iterations with different dataset splits was averaged to assess generalisability.

### Stage IV: bias analysis and mitigation

To check for fairness across ethnic groups, the optimal random forest model was analysed by ethnic groups. Particularly, this analysis refers to the performance range that is obtained by taking the difference between the maximum and minimum values for each metric across ethnic groups. For instance, if the FNR for an ML model has a maximum of 0.17 for the “White” ethnic group and a minimum of 0.51 for the “Asian” ethnic group, the performance range is calculated as follows:

$${\mathrm{Performance}\;\mathrm{range}}_{\mathrm{FNR}}=\max\{\mathrm{FNR}\}-\min\{\mathrm{FNR}\},$$

which equals 0.34 or 34%. The closer the performance range is to 0 the better the performance of the model. In this study, two bias mitigation techniques (reductions with EG and threshold optimiser) were evaluated to balance performance metrics across ethnic groups.

#### Reductions with exponentiated gradient

The reductions algorithm (22) was applied during training to limit performance ranges across ethnic groups. A fairness constraint was defined on FNR parity, requiring the FNR range between ethnic groups to be at most 0.2. The model was trained and evaluated to measure unfairness based on this constraint. The EG algorithm assigned weights to training instances that reduce the overall violation of the fairness constraint, with higher weights to instances contributing more to unfairness. The weighted training data was used to retrain the model to focus more on instances contributing to unfairness. These steps were repeated 10 times, updating instance weights iteratively to reduce bias while maintaining accuracy across ethnic groups. At each iteration, the algorithm updates the model parameters by considering the gradient of an objective function incorporating both predictive balanced accuracy and fairness constraints. The model parameters are then updated to effectively adjust the model to reduce bias while maintaining predictive balanced accuracy.

#### Threshold optimiser

To check for performance discrepancies across demographic groups, the post-processing threshold-optimiser approach (28) was utilised. Specifically, the threshold optimiser tuned the decision boundary of the random forest classifier to achieve parity in the balanced accuracy metric between ethnic groups subject to enforcing constraints on the FNRs per group. This optimised the fairness-balanced accuracy trade-off solution without needing to modify the underlying ML model or training procedure. The threshold optimiser takes an already trained model and fits a transformation function to the model’s outputs to satisfy certain fairness constraints. This approach allows for mitigating unfairness when developers have no control over the training process of the model, which may occur due to practical limitations or considerations around security or privacy.

## Results

This section presents the findings from the analysis of hospital admission records for patients with LD and MLTCs. The results are structured to address key research questions, including the primary and prevalent conditions for hospital admissions, the distribution and factors influencing LOS, and the application of ML models to predict LOS while addressing bias across ethnic groups. Insights from the study are further supported by performance evaluations of ML models and the application of bias mitigation techniques to improve fairness and generalisability.

### What were the primary conditions for hospital admissions of patients with LD?

**Figure 3:**
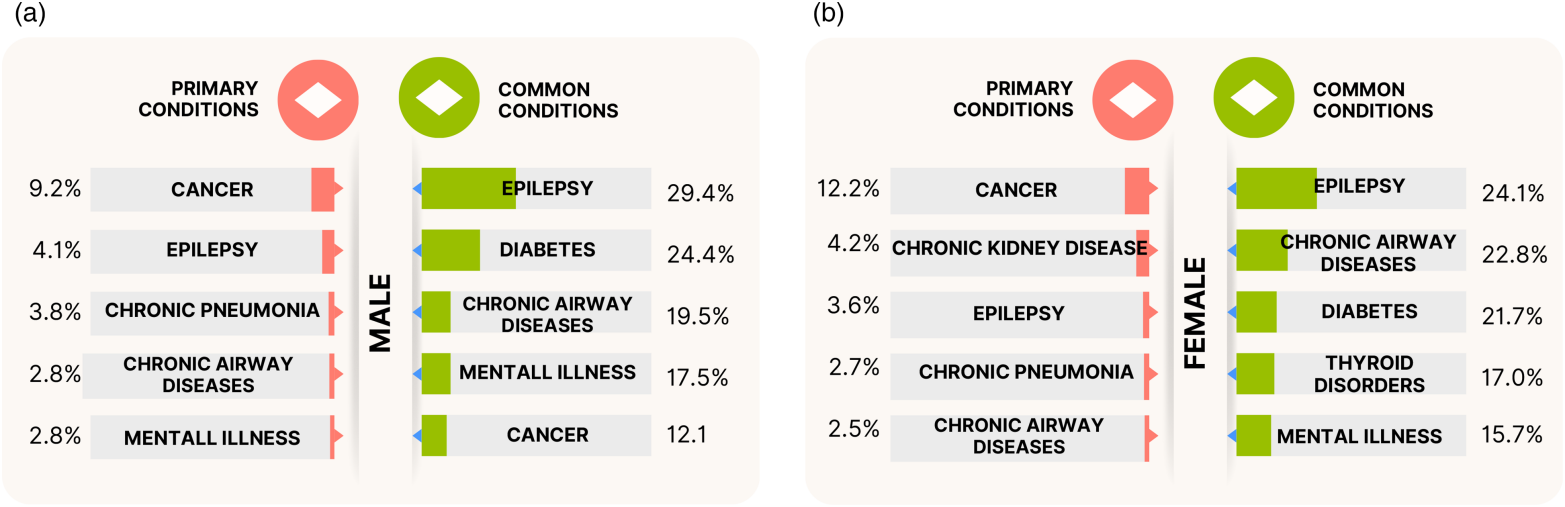
The top five primary conditions and top five prevalent (common) conditions treated or investigated during hospitalisations for the (**a**) males and (**b**) females with LD and MLTCs. The primary conditions indicate the main condition treated or investigated during the admission, and the prevalent conditions indicate the most frequently treated or investigated conditions (includes both primary and secondary diagnostic codes) across all unique hospitalisations of patients with LD and MLTCs.

The PEDW data includes a variable “diag_num”—a number used to identify the position of diagnosis assigned to a patient during a unique admission. Value “1” relates to the primary ICD-10 Diagnostic Code, which is the main condition treated or investigated during the relevant episode of healthcare. Values > 1 relate to secondary ICD-10 diagnostic codes. Analysis of 18,541 admissions of men and 17,587 admissions of women with LD from the last 10 years of study duration (January 2011–December 2021) showed cancer as the primary condition for admission, with 1,703 (9.2%) male and 2,149 (12.2%) female admissions. The subsequent top primary conditions differed by sex, respectively, as shown in Figure 3: epilepsy, chronic pneumonia, chronic airway diseases, and mental illness for males; vs. chronic kidney disease, epilepsy, chronic pneumonia, and chronic airway diseases for females. Supplementary Tables S14, S15 detail the top 10 primary conditions for both sexes including admission counts, patient numbers, and admission rates per patient. The high standard deviation (SD) values in the admission rate for some conditions indicate the variation in admission rates per individual, as some patients may be admitted more times than others. The admission rates are strongly influenced by the number of MLTCs across individuals.

### What were the prevalent conditions in hospital admissions for patients with LD?

Further 2011–2021 admission analysis was made on the PEDW data for the prevalent conditions treated or investigated during unique hospital admissions. This includes both primary and secondary diagnostic codes. The analysis revealed epilepsy as the most commonly treated LTCs during admissions, present in 29.4% of male and 24.1% of female admissions (Figure 3). The next most prevalent conditions in the male group were diabetes, chronic airway diseases, mental illness, and cancer, while the female group had chronic airway diseases, diabetes, thyroid disorders, and mental illness as the next most prevalent conditions after epilepsy. Figure 3 also indicates slightly higher rates of epilepsy, diabetes, and mental illness admissions in males compared to females, and higher rates of chronic airway diseases in females compared to males. Supplementary Table S16 provides the ranking of common LTCs treated during hospital admissions for the stated period.

### What is the distribution of the LOS across patients?

The LOS for all extracted patients’ hospitalisation records from birth ranged from a minimum of 0 day (indicating no overnight stay during admission) to over 5,000 days. The combined male and female group comprised 67,377 admissions. The median hospitalisation was $Q_{2}=2$ days with first and third quartiles of 0 day ($Q_{1}$) and 7 days ($Q_{3}$), respectively, giving an interquartile range (IQR) of $Q_{3}-Q_{1}$. This is illustrated using the box plot in Supplementary Figure S5, which includes lower and upper whiskers. All admissions with LOS days below the lower whiskers or above the upper whiskers are described as *outliers*. The lower whisker was the smallest LOS value in days greater than $Q_{1}-1.5\cdot IQR$, equal to $Q_{1}$ (0 day). The upper whisker was obtained as the largest LOS value in days less than $Q_{3}+1.5\cdot IQR$, obtained at 17 days.

#### Outliers

**Figure 4:**
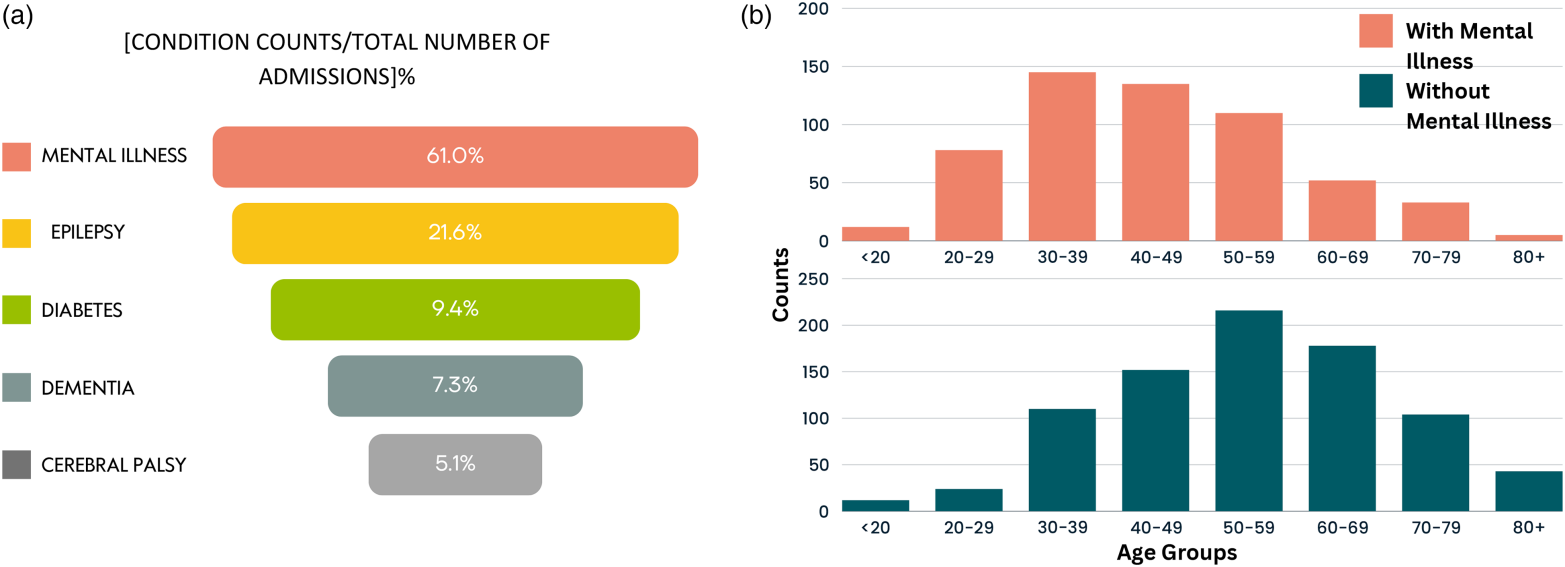
Distribution of patients with LOS ≥ 129 days. (**a**) The 4 most common conditions and (**b**) age distribution of patients with and without mental illness.

For the entire hospitalisation records of patients from birth, outliers (admissions with LOS >17 days) were further analysed to understand patterns among admissions with the most extended stays. Hence, quartile values from boxplots of outlier records were obtained: $Q_{1}=24$ days, $Q_{2}=36$ days, $Q_{3}=66$ days, and an IQR of 42 days. The upper whisker was the largest hospital stay under $Q_{3}+1.5\cdot \text{IQR}$, obtained at 129 days. Consequently, admissions with LOS ≥129 days were numerically evaluated. This amounted to 934 unique admissions. The majority of these admissions are related to mental illness. Generally, hospitalisations with very long LOS are common for mental health admissions, especially for those with challenging behaviours and autism/personality disorders posing safety risks (50, 51). Figure 4a further illustrates these findings, depicting the common conditions for stays ≥129 days. For these admissions with LOS ≥129, the most common condition was mental illness and epilepsy, followed by diabetes, dementia, and cerebral palsy. Supplementary Table S17 provides a full breakdown of related LTCs for admissions with LOS ≥129 days. Figure 4b further depicts, for all admissions with LOS ≥129 days, the age distribution for admissions involving mental illness and without mental illness. For the latter, age was normally distributed and skewed towards the older age groups. Given the study cohort includes patients with MLTCs, most patients admitted for a primary condition also experienced hospital episodes associated with other secondary conditions.

### What factors were prevalent in hospital stays ≥4 days?

Analysis was conducted prior to the ML predictions to identify common trends among admissions with LOS ≥4 days within the study duration. The extracted data was analysed by sex (male and female) across all admissions in the study duration. The one-sample chi-square and binomial tests were applied to all categorical features to test the null hypothesis that the variable categories occur with equal probability. Results stated in Supplementary Table S18 rejected this hypothesis for all categorical variables.

**Figure 5:**
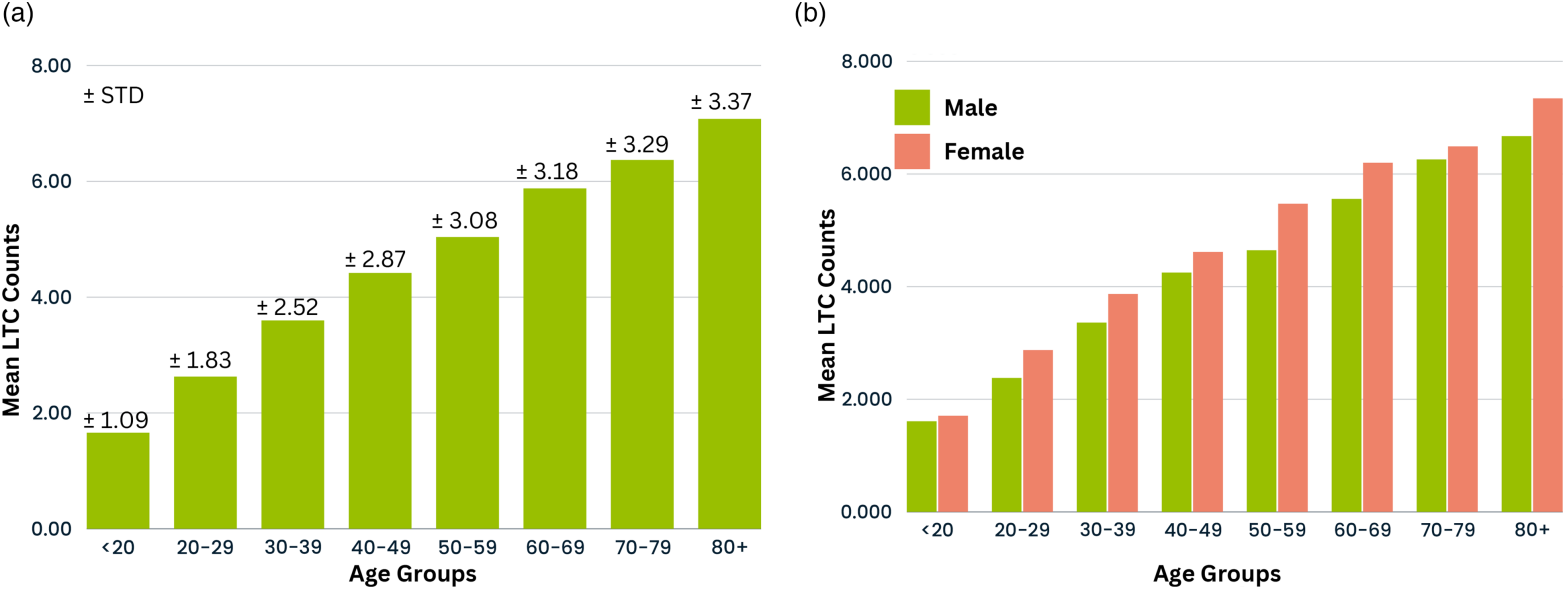
Distribution of mean MLTC count across age groups for (**a**) combined sexes and (**b**) individual sexes.

Excluding “unknown” groups, Supplementary Table S19 revealed ≥4 days stays were predominantly in patients aged ≥50 years, from more deprived socioeconomic quintiles, obese, and less physically active, compared to patients with short stays (LOS < 4 days). This observation was similar for both sexes. In addition, females with prescribed antipsychotic, antidepressant, or anti-manic/anti-epileptic medications were also seen to have more long stay admissions with LOS ≥4 days, compared to female admissions with LOS <4 days. Other factors influencing ≥4 days stays include the primary long-term condition for admission and MLTCs counts, which can increase hospital episodes in a single admission. Patients in this study cohort had between 1 and 21 comorbid conditions per person. Figures 5a,b illustrate the average MLTCs counts by age group for combined and separate sexes, showing a linear rise with age. Further analysis was also conducted on previous hospitalisation data that included the number of previous admissions and hospital episodes, cumulative hospital days from past admissions, and the number of MLTCs from previous admissions (see Supplementary Table S3). Patients with a higher number of MLTCs, cumulative hospital days from past admissions, and long-term conditions treated in previous admissions were more likely to be hospitalised for ≥4 days compared to patients with stays less than 4 days. Patients with more frequent prior admissions and higher counts of previous hospital episodes tended to have short LOS in their current admission. This is illustrated in Supplementary Figures S6–S9. Those patients with LOS <1 day were most likely to be attending routine appointments rather than emergency visits.

### Can machine learning predict the LOS across hospital admissions?

**Figure 6:**
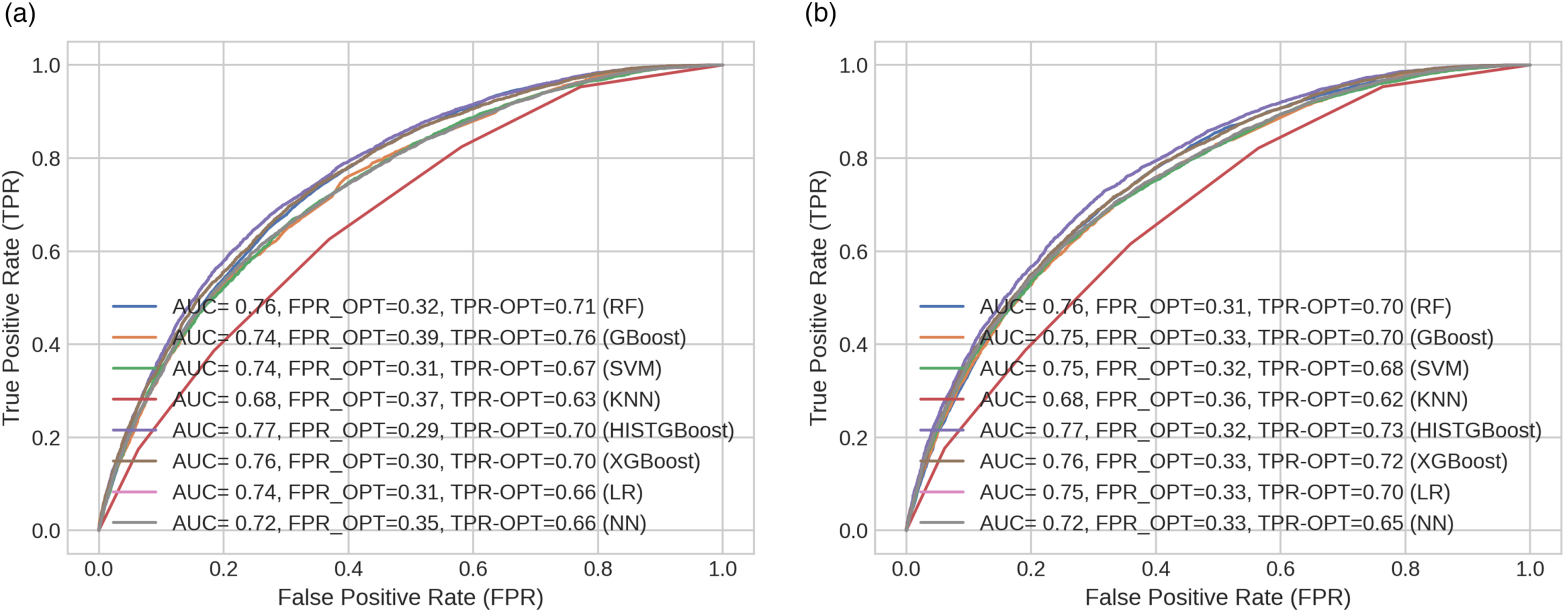
ROC curves across models indicating their optimal points (FPR and TPR) for (**a**) male and (**b**) female cohorts.

Several classification models were developed for the prediction of the LOS using the selected features described in Supplementary Table S3. The models were designed to predict whether a patient’s admission would have LOS <4 days or ≥4 days. Predictions were made using available patient data up until the first 24 h of admission. The classification performance of each model is presented in Supplementary Table S20. Additionally, Figures 6a,b depict the ROC curve for each classifier, along with the AUC and optimal ROC point values. An optimal ROC point refers to the point on an ROC curve that provides the best balance between the TPR and the FPR for a given classification model.

#### Classification performance

In this study, the HISTGBoost and RF classifiers showed the best performance compared to the other models as shown in Supplementary Table S20. The HISTGBoost classifier achieved higher AUC (M: 0.771, F: 0.773) and balanced accuracy (M: 0.701, F: 0.705). This was followed by the XGBoost classifier [AUC = 0.763 (M), 0.761 (F); balanced accuracy = 0.695 (M), 0.692 (F)] and the RF classifier [AUC = 0.759 (M), 0.756 (F); balanced accuracy = 0.690 (M), 0.689 (F)]. Regarding the FNR (indicating patients predicted to be discharged early when a longer (i.e., ≥4 days) hospital stay is required), the RF classifier returned lower values compared to the other models [FNR = 0.224 (M), 0.229 (F)]. The FNR value was approximately 7% lower for the RF classifier than the HISTGBoost model for the male group and ∼6% lower than the XGBoost model for the female group. The RF demonstrated optimal performance for both the male and female groups as shown by its low FNRs and high balanced accuracy. The low FNR indicates fewer missed cases where patients require longer hospitalisation ≥4 days, while the balanced accuracy shows high predictive performance.

#### Is the performance of the best model consistent across different train/test combinations?

The selected RF model was further evaluated using 10 randomly selected distinct train/test combinations to assess its performance across different samples. The mean performance (with SD) across all 10 iterations is provided in Supplementary Table S21 for the male and female cohorts. The mean value obtained for each parameter across sexes is similar to the performance detailed in Supplementary Table S20 for the male and female cohorts, and the SD is less than 0.007 for all parameters as shown in Supplementary Table S21. This suggests the RF model achieves consistent performance across different data samples, indicating it has good generalisability and is not overfitting on the given training set. The low SD also shows the model yields reliable and stable predictions.

### How did the best model perform across ethnic groups?

The RF model that showed optimal performance was further evaluated to address potential performance bias associated with the ethnic groups. Supplementary Tables S22, S23 provide an overview of the RF model’s performance segmented by ethnic groups for the male and female groups, respectively.

#### Males

For males, the “Other” ethnic group exhibited the highest FNR at 33.3% followed by the “Black” group with FNR of 27.3%. The “Black” group had the lowest balanced accuracy at 59.7%. In contrast, the “Asian” group demonstrated the lowest FNR of 19.5%, and the “White” group had the highest balanced accuracy of 69.2%. Overall, there was an approximate 13.8% range in the FNR and a 9.5% range in balanced accuracy across the considered ethnic groups. The model’s performance for the “White” ethnic group closely resembled the overall model performance (detailed in Supplementary Table S20 for the RF model), most likely because the majority of the training data (79%) originated from the “White” group. The “White” group constituted 79% of the entire male cohort.

#### Females

For females, the model’s performance for the “White” group closely matched the overall classifier performance, attributable to the high representation of the “White” group (80.6% of training, 80% of total data). The model underperformed in predicting LOS for the “Black” group with the lowest balanced accuracy (66.7%), and highest FPR (50%). Both the “Black” and “Other” groups had the lowest representation in the extracted data (0.15% and 0.19%, respectively), suggesting insufficient data for optimal modelling. The “Other” group, despite having a low representation of admission records, demonstrated improved performance with the lowest FNR (11.1%) and the highest balanced accuracy (77.8%). This suggests the classifier was able to effectively model outcomes for the “Other” ethnic group given the available training samples. Overall, for females, there was a performance range in the FNR, and balanced accuracy of approximately 12%, and 11.1%, respectively across ethnic groups.

### Can consistency in ML performance be achieved across ethnic groups?

In the context of this study, an ideally fair model would exhibit consistent performance in predicting the LOS across ethnic groups. To improve the fairness of the LOS prediction models, two bias mitigation algorithms: one post-processing (threshold optimiser) and one in-processing (reductions approach with exponentiated gradient) were empirically investigated. Both approaches aimed to minimise the range of each performance metric across the ethnic groups. Each bias mitigation algorithm was assessed and compared to the unmitigated model to determine its effectiveness.

#### In-processing (reductions with EG)

Supplementary Tables S24, S25 overview the EG reductions performance. This approach worked best in reducing the performance range of the FNR for the males by 9% compared to the unmitigated model. However, the performance range was not optimised for the FPRs and balanced accuracies for both sexes (see Supplementary Tables S26, S27).

#### Post-processing (threshold optimiser)

Supplementary Tables S24, S25 depict the performance of the threshold-optimised model. For females, compared to the unmitigated model, the threshold optimiser reduced the FNR range across ethnic groups by 3.6% (see Supplementary Table S27). However, the range for the FPR and balanced accuracy increased by 9.8% and 5.6%, respectively across ethnic groups compared to the unmitigated model. Specifically, the optimiser did not substantially improve the model fairness across the female group. The threshold optimiser yielded better performance in males, reducing the range for the FPR and the balanced accuracy values across ethnic groups by 7.4% and 1.8%, respectively. However, the range for FNR increased slightly by 1.7% for the threshold optimiser compared to the unmitigated model. Particularly, the unmitigated classifier had a lower FNR range across the ethnic groups than the optimiser for males (see Supplementary Table S26).

In summary, although the fairness goal of equal performance across ethnic groups was not fully met, the post-processing threshold optimiser approach was more effective at improving performance uniformity across ethnic groups compared to the reduction with exponentiated gradient.

## Discussion

Prolonged hospital stays pose significant patient risks, including increased susceptibility to infections, falls, sleep deprivation, and physical and mental decline. To address these issues, NHS England’s Reducing Length of Stay (RLoS) program (52) aims to improve patient care and optimise resource use by minimising unnecessary delays in hospital discharges. The program’s national goal to reduce hospital stays of 21+ days by 25%–40% highlights the importance of timely discharge planning in improving patient outcomes, increasing capacity in urgent and emergency care, and freeing up hospital beds. However, individuals with LD experience poorer health outcomes compared to the general population, which often leads to prolonged hospital stays (10). Accurately predicting the LOS for this population is therefore crucial for optimising resource allocation and preventing unnecessarily prolonged or premature discharges (12).

This study contributes to these goals by developing ML models to predict hospital LOS for patients with LD and MLTCs while addressing fairness concerns across ethnic groups. An overview of findings from this study is detailed in Box 1. As ML increasingly guides healthcare decision-making, ensuring algorithmic fairness is critical to avoid exacerbating existing health disparities. However, the dataset used in this study exhibited imbalanced representation across ethnic groups, with underrepresentation of minority patients and missing ethnic information for a significant proportion of records. These imbalances resulted in performance disparities in the Random Forest (RF) model, particularly in FNRs for minority females, indicating poorer predictions of long stays for this subgroup.

**Box 1 Overview of findings**

• Analysed electronic health records of 9,618 patients with LD and MLTCs in Wales, examining 62,243 hospital admissions.
• Cancer was the top primary condition for hospital admissions in both males and females with LD. Epilepsy was the most commonly co-occurring condition across all admissions between January 2011 and December 2021.
• Hospital stays lasted a median of 2 days, with an IQR of 0–7 days. Stays exceeding 129 days were commonly related to mental illness.
• Common factors associated with patients with hospital stays ≥4 days included: age ≥50 years, higher socioeconomic deprivation, obesity, low physical activity as well as a higher number of MLTCs, cumulative hospital days from past admissions, and long-term conditions treated during previous admissions.
• A random forest machine learning model achieved AUCs of 0.759 (males) and 0.756 (females) in predicting the length of stay using data up to the first 24 h of admission.
• Before bias mitigation, the model demonstrated performance discrepancies across ethnic groups. Two bias mitigation approaches were tested, with the threshold optimiser outperforming the reductions approach in minimising some performance differences across groups.

To address these challenges, two bias mitigation techniques were applied: the threshold optimizer (post-processing) and reductions method with exponentiated gradient (in-processing). These techniques successfully reduced performance discrepancies across ethnic groups while maintaining strong overall predictive accuracy. However, this study underscores the critical importance of improving the completeness and consistency of data recording. Enhancing the quality of such data would provide more representative training examples, improving both the fairness and reliability of ML models and ultimately enabling more equitable healthcare outcomes.

In summary, this study advances the use of ML for LOS prediction by integrating fairness-focused methodologies, thereby contributing to equitable healthcare delivery for disadvantaged populations. These findings demonstrate the potential of ML models to enhance care planning, optimise resource allocation, and align with broader policy initiatives, such as the RLoS program. Future research should explore the scalability of these methods across different healthcare systems and address data gaps to further improve fairness and generalisability.

### Limitations

While the techniques employed in this study show promise, challenges remain in achieving a balance between fairness and accuracy, as highlighted by the results, addressing intersectional biases, and ensuring generalisability across diverse patient populations. Healthcare datasets are often constrained by privacy concerns and small sample sizes, further complicating the implementation of effective bias mitigation strategies. The dataset of Welsh patients used in this study exhibited significant imbalances in ethnic group representation, limiting the model’s ability to generalise and contributing to performance disparities across groups. Additionally, missing and incomplete data, categorised as “unknown” during preprocessing, may have obscured important nuances or introduced biases. Future research should focus on acquiring complete and consistent datasets, particularly for sensitive attributes such as ethnicity and socioeconomic status, to enhance model fairness and accuracy. Additionally, consistent annual health checks for the LD cohort could enhance data integrity and the accuracy of time-varying predictors such as BMI, smoking status, alcohol intake, and medication history (53).

### Future research directions

Future research should focus on expanding datasets to include larger and more diverse cohorts, enhancing applicability across regions, healthcare systems, and population groups. Incorporating longitudinal data and social determinants of health, such as income, education, living composition (e.g., whether a patient with LD lives alone or with a carer), and communication or mobility difficulties, could further refine predictions and improve utility for this cohort. Addressing intersectional biases based on overlapping attributes (e.g., age, ethnicity, and socioeconomic status) and developing advanced fairness algorithms to balance equity and accuracy remain essential. For real-world implementation, integrating the models into clinical decision support systems for adults with LD and conducting pilot studies in hospitals are key steps to evaluating usability and effectiveness. Training clinicians and administrators to interpret AI-driven predictions will be crucial for fostering trust and adoption. Lastly, extending the binary classification of hospital stays (short vs. long) into more granular ordinal or continuous prediction models could provide deeper insights into healthcare needs and outcomes for the study cohort.

## Data Availability

The datasets presented in this article are not readily available as all proposals to use SAIL data are subject to review by the independent IGRP. The anonymised individual-level data sources used in this study are available in the SAIL Databank at Swansea University, Swansea, UK, Before any data can be accessed, approval must be given by the IGRP. The IGRP gives careful consideration to each project to ensure proper and appropriate use of SAIL data. When access has been granted, it is gained through a privacy-protecting safe haven and remote access system referred to as the SAIL Gateway. SAIL has established an application process to be followed by anyone who would like to access data via SAIL at: https://www.saildatabank.com/application-process.
