# Supplementary material for: Equitable hospital length of stay prediction for patients with learning disabilities and multiple long-term conditions using machine learning
Source: Front Digit Health. 2025 Feb 14;7:1538793. doi: 10.3389/fdgth.2025.1538793 (PMC11868268; doi:10.3389/fdgth.2025.1538793)
Supplement: Supplementary file 1 [file Datasheet1.pdf]

SUPPLEMENTARY FILES

Table S1 List of LTCs considered in this study.

CONDITIONS
ANAEMIA
BARRETTS OESOPHAGUS
BRONCHIECTASIS
CANCER
CARDIAC ARRHYTHMIAS
CEREBRAL PALSY
CHRONIC CONSTIPATION
CHRONIC DIARRHOEA
CHRONIC AIRWAY DISEASES
CHRONIC ARTHRITIS
CHRONIC PAIN CONDITIONS
CHRONIC PNEUMONIA
CIRRHOSIS
CHRONIC KIDNEY DISEASE
CORONARY HEART DISEASE
DEMENTIA
DIABETES
DYSPHAGIA
EPILEPSY
HEARING LOSS
HEART FAILURE
HYPERTENSION
INFLAMMATORY BOWEL DISEASE (IBD)
INSOMNIA
INTERSTITIAL LUNG DISEASE
MENOPAUSAL AND PRE-MENOPAUSAL
MENTAL ILLNESS
MS
NEUROPATHIC PAIN
OSTEOPOROSIS
PARKINSONS
POLYCYSTIC OVARY SYNDROME

Table S1 continued from previous page

CONDITIONS
PSORIASIS
Peripheral Vascular Disease (PVD)
REFLUX DISORDERS
STROKE
THYROID DISORDERS
TOURETTE
VISUAL IMPAIRMENT

Table S2 Demographic description of the cohort.

	Male			Female		
	Patients (%)	Unique Admissions (%)	Long-stay rate %	Patients (%)	Unique Admissions (%)	Long-stay rate %
Total	4929	32275	38.885	4689	29968	38.798
Age						
<30	159	1565	36.741	119	1525	37.967
30-39	594 (12.051)	4883 (15.129)	32.521	531 (11.324)	4171(13.918)	33.277
40-49	1065 (21.607)	6954 (21.546)	35.620	965 (20.58)	6103(20.365)	32.591
50-59	1229 (24.934)	7677 (23.786)	38.205	1083 (23.097)	6840(22.824)	35.146
60-69	1025 (20.795)	6556 (20.313)	42.145	934 (19.919)	6361(21.226)	38.610
70-79	605 (12.274)	3631 (11.25)	44.643	640 (13.649)	3404(11.359)	54.083
80+	252 (5.113)	1009 (3.126)	58.771	417 (8.893)	1564(5.219)	62.084
Ethnic group						
Asian	79 (1.603)	576 (1.785)	46.528	64 (1.365)	564 (1.882)	35.461
Black	19 (0.385)	75 (0.232)	52.000	14 (0.299)	44 (0.147)	65.909
Other	19 (0.385)	76 (0.235)	34.211	18 (0.384)	56 (0.187)	30.357
Unknown	1225 (24.853)	6208 (19.235)	39.030	1124 (23.971)	5253 (17.529)	40.034
White	3587 (72.773)	25340 (78.513)	38.650	3469 (73.982)	24051 (80.256)	38.576
WIMD						
1 (Most Deprived)	1227 (24.893)	9071 (28.105)	37.813	1207 (25.741)	8177 (27.286)	38.963

Table S2 continued from previous page

	Male			Female		
	Patients (%)	Unique Admissions (%)	Long-stay rate %	Patients (%)	Unique Admissions (%)	Long-stay rate %
2	990 (20.085)	6947 (21.524)	38.491	943 (20.111)	6855 (22.874)	35.799
3	791 (16.048)	4943 (15.315)	41.958	770 (16.421)	4660 (15.55)	42.253
4	721 (14.628)	4184 (12.964)	41.276	700 (14.929)	4433 (14.792)	39.274
5 (Least Deprived)	511 (10.367)	3068 (9.506)	39.309	494 (10.535)	2811 (9.38)	39.772
Unknown	689 (13.978)	4062 (12.586)	35.426	575 (12.263)	3032 (10.117)	38.226

Remark: Long-stay rate defined in formula (1).

The 'Patients' column across age groups takes the latest age group for each unique patient.

Table S3 Variables used for the Machine Learning (ML) training and analysis.

	**Variable name**	**Description**	**Variable type**
		Input variables to ML models	
Patient lifestyle and history	BMI	BMI value documented closest to the admission date	Categorical Variable: (pre-obesity, obesity class I, obesity class III, normal weight, underweight, and 'Unknown')
	SMOKING_HISTORY	History of smoking prior to admission	Categorical Variable (Yes, No, 'Unknown')
	ALCOHOL_HISTORY	history of alcohol intake prior to admission	Categorical Variable (Yes, No, 'Unknown')
	PHYSICAL	Patient does light/regular exercise	Categorical Variable (Yes, No, 'Unknown')
	AUTISM	Indicates if patient is autistic	Binary Variable (Yes/No)
Prior hospitalisation data	NUM_PRVADMISSION_1RY	Number of hospital admissions in the past year from admission date	Numeric variable
	NUM_PRVEPISODES_1RY	Total hospital episodes from all 1 year prior admissions	Numeric variable
	NUM_PRVCOMORBID_1RY	Frequency of LTCs during previous admissions over past 1 year	Numeric variable
	NUM_PRVADMISSION_3RY	Number of hospital admissions in the past 3 years from admission date	Numeric variable
	NUM_PRVEPISODES_3RY	Total hospital episodes from all 3 years prior admissions	Numeric variable
	NUM_PRVCOMORBID_3RY	Frequency of LTCs during previous admissions over past 3 years	Numeric variable

Table S3 – continued from previous page

	Variable name	Description	Variable type
	NUM_PRVHOSPITAL_DAYS_1YR	Cumulative hospital days 1 year prior to admissions	Numeric variable
	NUM_PRVHOSPITAL_DAYS_3YR	Cumulative hospital days 3 years prior to admissions	Numeric variable
Prescriptions	MEDICATIONS	History of antipsychotic, antidepressant or anti-manic / anti-epileptic medications	Binary variable (Yes/No)
Admission data	TOTAL_COMORBIDITY	Total number of multiple LTC by patient as at 24 hours after admission date	Numeric variable
	NUMEPISODES_24HRS	Total number of clinical episodes within 24 hours of admission	Numeric variable
	NUMCOMORBIDITIES_24HRS	Number of LTCs linked to hospital episodes in the first 24 hours of admission	Numeric variable
	COND	Indicates which of the 64 LTCs were linked to the patients' episodes in the first 24 hours of admission	Binary Variable for each condition. 60 LTCs for males and 62 for females
		Target variable	
Target variable	LOSClass	Indicator of LOS	Binary Variable 0: LOS<4 and 1: LOS $\geq$ 4
		Demographic variables (for analysis)	
Demographics	AGEGRP_AT_ADMIS_DT	Age group of patient as at admission date	Categorical Variable (see table S2)
	Ethnic group	Ethnic group of patient	Categorical Variable (see table S2)
	WIMD	Welsh Index of Multiple Deprivation version 2019	Numeric variable on a scale of 1 (most deprived) to 5 (least deprived), and 'Unknown' group

Remark: 'AUTISM' is a neurodevelopmental disorder stated to occur from birth(54) but may be undiagnosed until adulthood. Therefore, this variable takes the value 1 for all the admission records of an autistic patient, regardless of when they were first diagnosed with autism.

Table S4 Mortality statistics of the cohort of hospitalised patients for the male and female sexes.

	Male		Female	
	Mortality	In-hospital mortality	Mortality	In-hospital mortality
Total	1904	979	1777	850

Table S4 continued from previous page

	Male		Female	
	Mortality	In-hospital mortality	Mortality	In-hospital mortality
Age				
<30	26	10	12	5
30-39	82	31	66	29
40-49	231	111	196	76
50-59	443	205	337	147
60-69	517	281	453	237
70-79	395	227	381	191
80+	210	114	332	165
WIMD				
1 (Most Deprived)	452	225	437	216
2	371	209	378	187
3	353	181	312	143
4	288	153	298	133
5 (Least Deprived)	196	104	180	90
Unknown	244	107	172	81

Table S5 List of antipsychotic, antidepressant, and anti-manic/ anti-epileptic medications considered in this study.

MEDICATIONS
EPILIM
TEGRETOL
LAMOTRIGINE
LEVETIRACETAM
CARBAMAZEPINE
SODIUM VALPROATE
PHENYTOIN
TOPIRAMATE
GABAPENTIN
EPANUTIN

Table S5 continued from previous page

**MEDICATIONS**
CLOBAZAM
CLONAZEPAM
PHENOBARBITAL
DEPAKOTE
PREGABALIN
KEPPRA
LAMICTAL
PRIMIDONE
VALPROIC ACID
PROCHLORPERAZINE
ZONISAMIDE
TOPAMAX
VIGABATRIN
LACOSAMIDE
MYSOLINE
RIVOTRIL
EPISENTA
OXCARBAZEPINE
RUFINAMIDE
ACETAZOLAMIDE
ETHOSUXIMIDE
PERAMPANEL
ESLICARBAZEPINE ACETATE
SABRIL
ZONEGRAN
LYRICA
FLUOXETINE HYDROCHLORIDE
NEURONTIN
ZARONTIN
TIAGABINE
ON LITHIUM
TRILEPTAL
DESITREND
ORLEPT

Table S5 continued from previous page

**MEDICATIONS**
VIMPAT
MIRTAZAPINE
THIORIDAZINE
ZEBINIX
TAPCLOB
PAROXETINE HYDROCHLORIDE
FRISIUM
QUETIAPINE
DIAMOX
VENLAFAXINE
SERTRALINE HYDROCHLORIDE
CONVULEX
ESLICARBAZEPINE
RETIGABINE
CARBAGEN SR
EPIMAZ
LEVOMEPROMAZINE
TRAZODONE HYDROCHLORIDE
CLOMIPRAMINE HYDROCHLORIDE
AMITRIPTYLINE HYDROCHLORIDE [ANTIDEPRESSANT]
ZONISAMIDE
DOSULEPIN HYDROCHLORIDE
PROMAZINE HYDROCHLORIDE
OLANZAPINE
CHLORPROMAZINE HYDROCHLORIDE
HALOPERIDOL [ANTIPSYCHOTIC]
INOVELON
AMISULPRIDE
PHENOBARBITONE SODIUM
GABITRIL
FYCOMPA
TERIL
EMESIDE
PALIPERIDONE

Table S5 continued from previous page

MEDICATIONS
DULOXETINE
RISPERIDONE

CATEGORISATION FOR ALCOHOL STATUS PER PATIENT

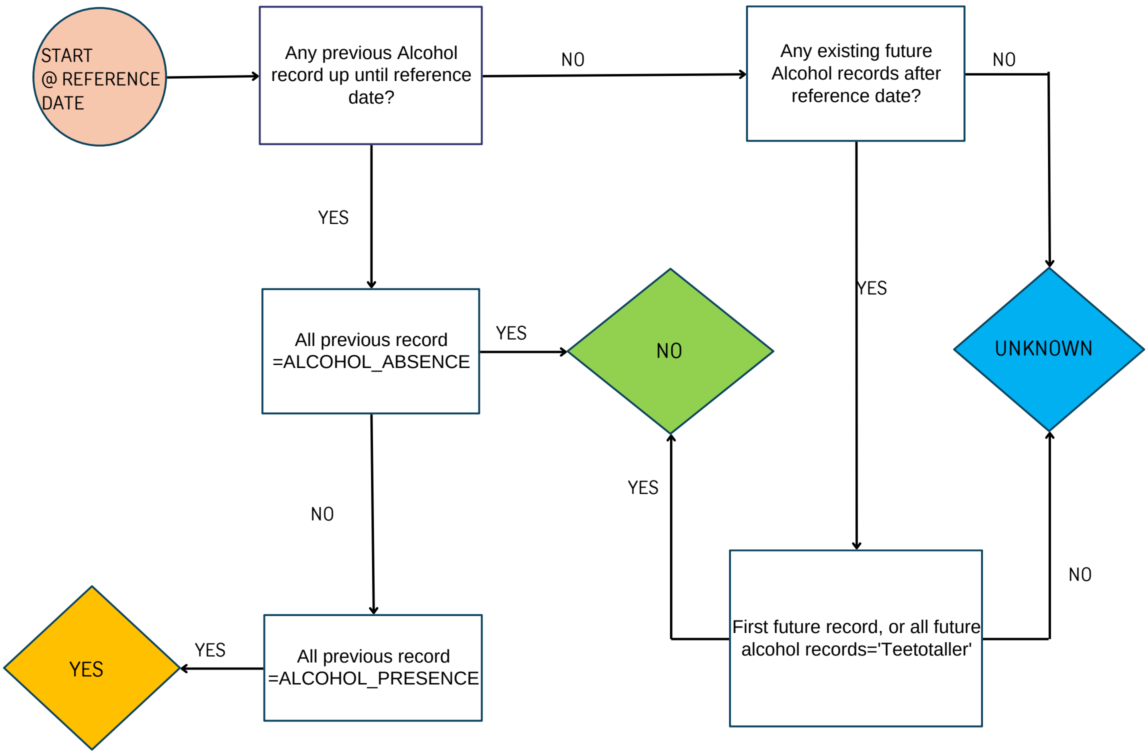

Figure S1. Flow chart diagram describing the algorithm for alcohol status at admission (reference date in the figure represents the admission date).

To categorise the alcohol consumption history across patients as depicted in Figure S1, the following read code and ICD-10 code descriptions were classified into several sets as follows:

- NON_DRINKER=['Teetotaller']
- CURRENT_NON_DRINKER=['Current non drinker']
- MODERATE_DRINKER=['Ex-light drinker - (1-2u/day)', 'Drinks beer and spirits', 'Ex-trivial drinker (<1u/day)', 'Trivial drinker - <1u/day', Social drinker', 'Alcohol intake within recommended sensible limits', 'Beer drinker', 'Light drinker', 'Drinks wine', 'Moderate drinker', 'Spirit drinker']

- HEAVY_DRINKER=['Mental and behavioural disorders due to use of alcohol','[X]Mental and behavioural disorders due to use of alcohol: psychotic disorder', 'Chronic alcoholism in remission','Alcohol-induced chronic pancreatitis', '[X]Mental and behavioural disorders due to use of alcohol: harmful use','[X]Mental and behavioural disorders due to use of alcohol', 'Alcohol withdrawal delirium','[X]Alcohol withdrawal-induced seizure','Chronic alcoholism NOS','Alcoholic hepatitis', 'Under care of community alcohol team','[X]Mental and behavioural disorders due to use of alcohol: dependence syndrome', 'Continuous chronic alcoholism','Alcohol dependence syndrome NOS','Alcoholic fatty liver','Very heavy drinker','Chronic alcoholism', 'Alcoholic liver damage unspecified','Very heavy drinker - >9u/day','Binge drinker','Alcohol intake above recommended sensible limits', 'Alcohol abuse monitoring','Hazardous alcohol use','Heavy drinker','Harmful alcohol use','Alcoholic cirrhosis of liver', 'Alcohol misuse','Acute alcoholic intoxication in alcoholism',]
- DRINKER=['Feels should cut down drinking', 'Declined referral to specialist alcohol treatment service', 'Alcohol dependence syndrome', 'Current non drinker', 'H/O: alcoholism', 'Alcohol withdrawal syndrome',]
- FORMER_DRINKER=['Ex-light drinker - (1-2u/day)','Ex-heavy drinker - (7-9u/day)','Ex-very heavy drinker-(>9u/d)', 'Ex-trivial drinker (<1u/day)']
- ALCOHOL_ABSENCE=NON_DRINKER + MODERATE_DRINKER+ CURRENT_NON_DRINKER
- ALCOHOL_PRESENCE= HEAVY_DRINKER + DRINKER + FORMER_DRINKER

CATEGORISATION FOR SMOKING HISTORY PER PATIENT

To categorise the smoking history across patients as depicted in Figure S2, the following readcode descriptions were classified into several sets as follows:

- NON_SMOKER=['Never smoked tobacco','Current non-smoker']
- EX_SMOKER=['Ex roll-up cigarette smoker','Ex cigar smoker','Ex pipe smoker','Ex-very heavy smoker (40+/day)', 'Ex-cigarette smoker','Ex-heavy smoker (20-39/day)','Ex smoker', 'Current non-smoker','Stopped smoking', 'Ex-smoker - amount unknown']
- SMOKER=['Smoking status at 52 weeks','Smoking restarted','Smoking status between 4 and 52 weeks','Smoking status at 4 weeks', 'Current smoker annual review - enhanced services administration','Minutes from waking to first tobacco consumption', 'Smoking cessation programme start date','Recently stopped smoking','Smoking free weeks','Smoking reduced', 'Negotiated date for cessation of smoking','Smoking started','Keeps trying to stop smoking','Failed attempt to stop smoking', 'Smoking cessation milestones','Not a passive smoker','Ready to stop smoking','Passive smoker','Thinking about stopping smoking', 'Pipe smoker','Trivial smoker - < 1 cig/day','Cigarette smoker','Current smoker','Rolls own cigarettes','Trying to give up smoking', 'Not interested in stopping smoking']
- SMOK_UNKNOWN=['Refusal to give smoking status','Tobacco consumption unknown']
- SMOKED=SMOKER +EX_SMOKER

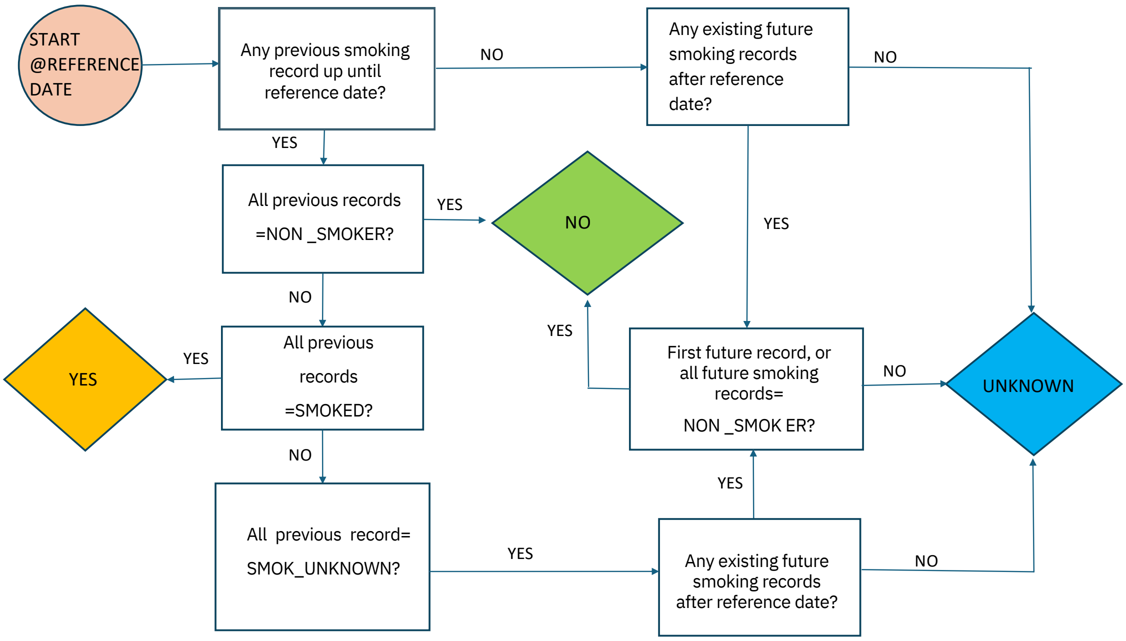

Figure S2. Flow chart diagram describing the algorithm for coding smoking history at admission (reference date in the figure represents the admission date).

Table S6 Results of normality tests applied to the dataset; KS denotes the Kolmogorov Smirnov test. The threshold for P-value is 0.05, hence, for P-value> 0.05, the normality test is positive otherwise, the independent variable is not normally distributed.

	Male		Female	
	KS Normality Test		KS Normality Test	
	Stat	P-value	Stat	P-value
NUM_PRVEPISODES_1YR	0.354	0.000	0.353	0.000
NUM_PRVCOMORBID_1YR	0.222	0.000	0.217	0.000
NUM_PRVCOMORBID_3YR	0.188	0.000	0.180	0.000
NUM_PRVEPISODES_3YR	0.366	0.000	0.369	0.000
NUMEPISODES_24HRS	0.269	0.000	0.264	0.000
NUMCOMORBIDITIES_24HRS	0.324	0.000	0.313	0.000

Table S6 continued from previous page

	Male		Female	
	KS Normality Test		KS Normality Test	
	Stat	P-value	Stat	P-value
NUM_PRVADMISSION_1YR	0.397	0.000	0.399	0.000
NUM_PRVADMISSION_3YR	0.401	0.000	0.412	0.000
NUM_PRVHOSPITAL_DAYS_1YR	0.351	0.000	0.355	0.000
NUM_PRVHOSPITAL_DAYS_3YR	0.345	0.000	0.349	0.000
TOTAL_COMORBIDITY	0.144	0.000	0.139	0.000

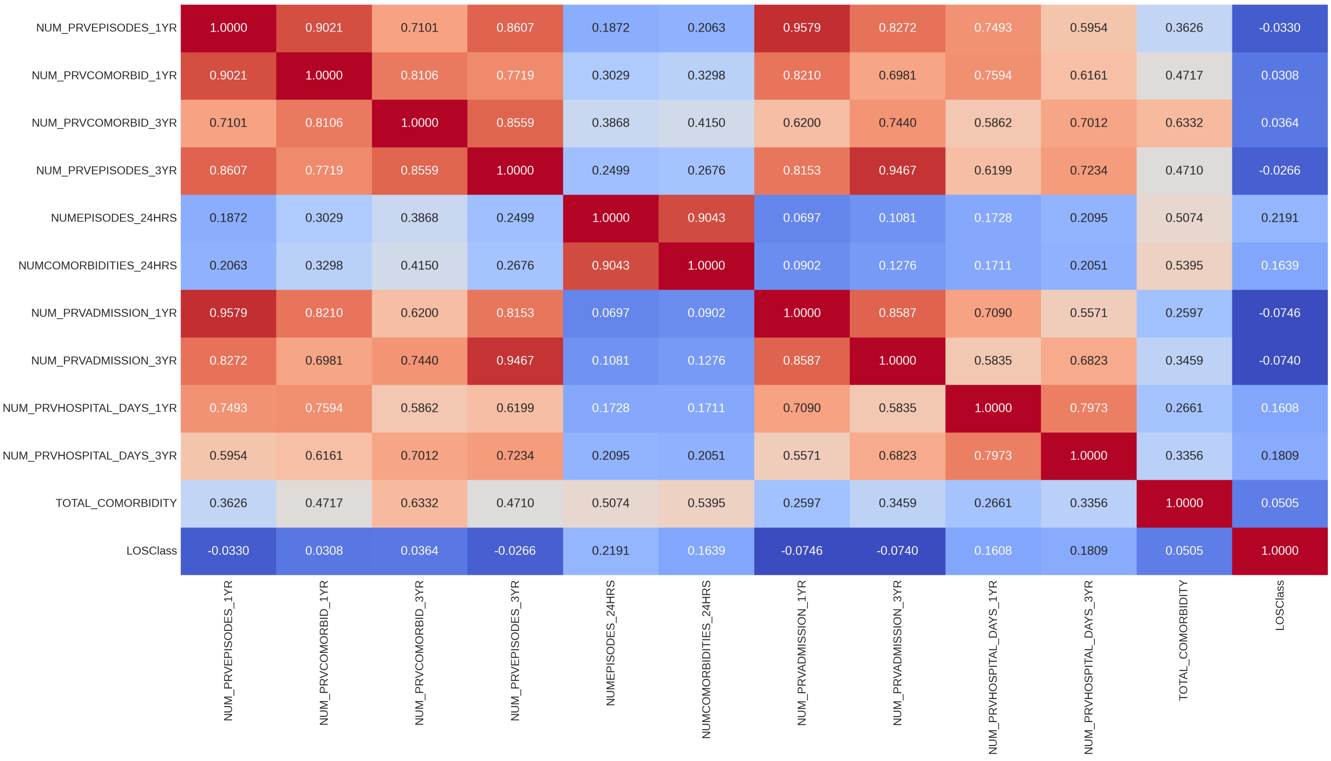

Figure S3. Feature correlation for males.

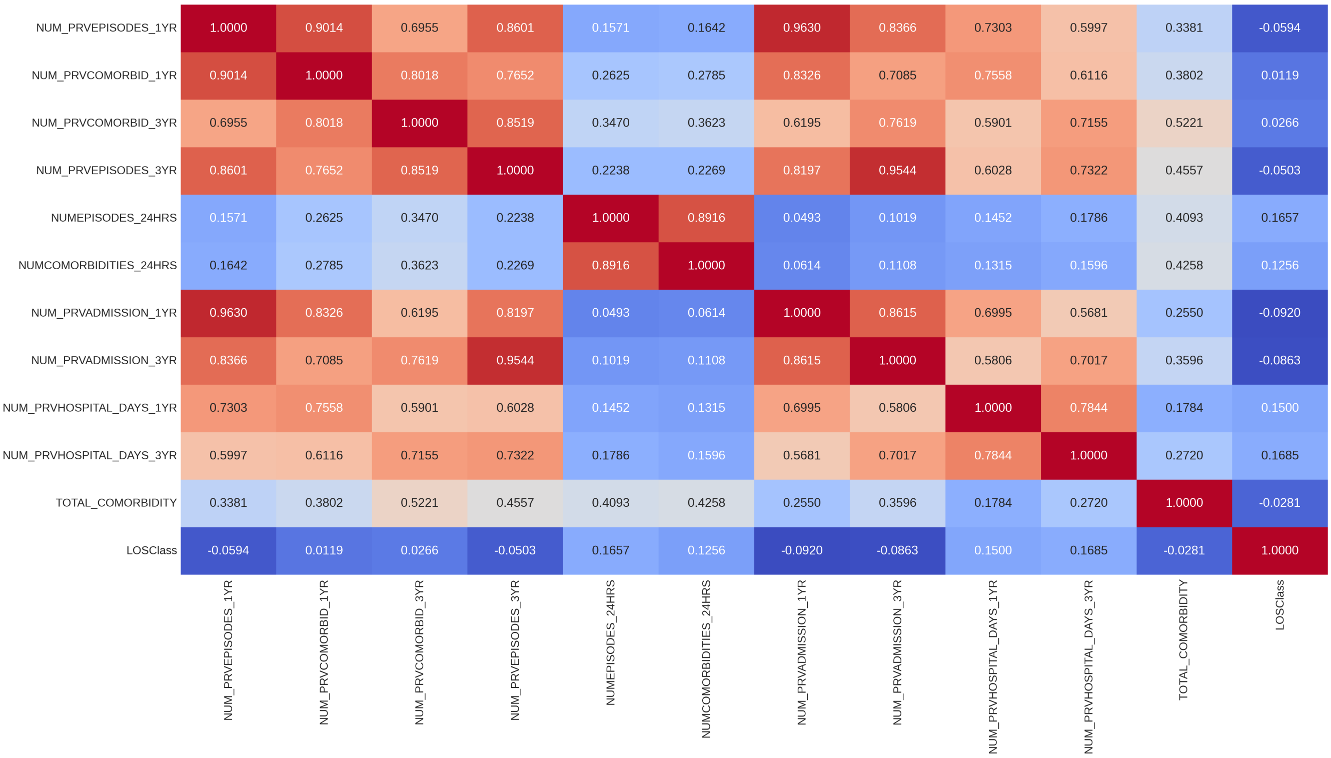

Figure S4. Feature correlation for females.

Table S7. Raw Variable.

Admission ID	PHYSICAL
001	Yes
002	No
003	Unknown

Table S8. One-hot encoded variable.

Admission ID	PHYSICAL_Yes	PHYSICAL_No	PHYSICAL_Unknown
001	1	0	0
002	0	1	0
003	0	0	1

Table S9 Demographic distribution for training and validation sets for male cohort.

	Training			Test		
	Unique Admissions (%)	LOS 0	LOS 1	Unique Admissions	LOS 0	LOS 1
Total	12550	6275	6275	16138	9863	6275
Age						
<20	41	29	12	38	25	13
20-29	564	295	269	751	470	281
30-39	1865	1051	814	2412	1638	774
40-49	2644	1419	1225	3493	2241	1252
50-59	3017	1537	1480	3816	2363	1453
60-69	2580	1209	1371	3306	1914	1392
70-79	1446	627	819	1787	985	802
80+	393	108	285	535	348	227
Ethnic groups						
Asian	239	99	140	276	148	128
Black	35	18	17	37	15	22
Other	31	20	11	41	26	15
Unknown	2365	1135	1230	3110	1917	1193
White	9880	5003	4877	12674	7757	4917
WIMD						
1 (Most Deprived)	3529	17820	1747	4489	2806	1683
2	2656	1341	1315	3540	2181	1359
3	1959	939	1020	2458	1404	1054
4	1640	765	875	2101	1249	852
5 (Least Deprived)	1232	610	622	1521	937	584
Unknown	1534	838	696	2029	1286	743

Table S10 Demographic distribution for training and validation sets for female cohort.

	Training			Test		
	Unique Admissions	LOS 0	LOS 1	Unique Admissions	LOS 0	LOS 1
Total	11626	5813	5813	14984	9170	5814
Age						
<20	31	16	15	33	22	11
20-29	571	282	289	708	445	263
30-39	1595	915	680	2101	1393	708
40-49	2231	1251	980	3099	2090	1009
50-59	2614	1432	1182	3428	2206	1222
60-69	2435	1216	1219	3203	1966	1237
70-79	1460	517	943	1661	763	898
80+	689	184	505	751	285	466
Ethnic group						
Asian	207	109	98	274	172	102
Black	19	<5	*	18	6	12
Other	27	*	*	27	18	9
Unknown	2002	945	1057	2613	1567	1046
White	9371	4738	4633	12052	7407	4645
WIMD						
1 (Most Deprived)	3195	1598	1597	4090	2501	1589
2	2589	1357	1232	3455	2233	1222
3	1833	862	971	2374	1376	998
4	1766	889	877	2185	1321	864
5 (Least Deprived)	1109	559	550	1369	801	568
Unknown	1134	548	586	1511	938	573

Additional cells have been masked to prevent disclosure

Table S11 Parameter configurations for all classification models.

Classification models	Parameters	Value
LR	C	1.0
	Max_iter	1000
	penalty	l2
	Random_state	none
	Multi_class	auto
RF	Max_depth=2	None
	Random_state=0	None
	N_estimators	100
	Max_samples	None
	Max_features	sqrt
	criterion	gini
KNN	N_neighbors	5
	Weights	Uniform
	Metric=euclidean	Euclidean (i.e., minkowski)
	p	2
	Leaf_size	30
GBoost	Learning_rate	0.1
	N_estimators	100
	Max_depth	1
	Min_samples_split	2
	Min_samples_leaf	1
	Random_state	0
	subsample	1.0
	loss	Log_loss
	criterion	Friedman_mse
SVM	C	1.0
	kernel	linear
	gamma	scale
	degree	3
	Probability	True
	Max_iter	-1

Table S11 continued from previous page

Classification models	Parameters	Value
HISTGBoost	Max_bins	255
	Max_iter	100
	loss	Log_loss
	Learning_rate	0.1
	Max_depth	None
XGBoost	Learning rate	None
	Max_depth	None
	Eval_metric	'mlog_loss'
	N_estimators	100

Remark: Other parameters not included in this table take in default values set by the sklearn ML library.

Table S12 Parameter summary for the sequential NN with the male cohort.

Layer (type)	Output shape	Param #	Tr. Param #
Linear-1	[11578, 75]	5 700	5 700
ReLU-2	[11578, 75]	0	0
Linear-3	[11578, 2]	152	152
Total params: 5 852			
Trainable params: 5 852			
Non-trainable params: 0			

Table S13 Parameter summary for the sequential NN with the female cohort.

Layer (type)	Output shape	Param #	Tr. Param #
Linear-1	[10732, 78]	6 162	6 162
ReLU-2	[10732, 78]	0	0
Linear-3	[10732, 2]	158	158
Total params: 6 320			
Trainable params: 6 320			

Non-trainable params: 0

Table S14 Ranking of top 10 primary conditions treated during hospitalisation between January 2011 to December 2021, for males with LD.

CONDITION	ADMISSION COUNT	PATIENT COUNT	MEAN ADMISSIONS PER PATIENT	SD OF ADMISSIONS PER PATIENT	MEDIAN ADMISSIONS PER PATIENT	MODE ADMISSIONS PER PATIENT
CANCER	1703	349	4.880	7.567	2	1
EPILEPSY	764	328	2.329	2.599	1	1
CHRONIC PNEUMONIA	713	247	2.887	1.775	3	2
CHRONIC AIRWAY DISEASES	519	178	2.916	5.897	1	1
MENTAL ILLNESS	510	193	2.642	2.880	1	1
DIABETES	334	145	2.303	2.462	1	1
CORONARY HEART DISEASE	312	176	1.773	1.289	1	1
CHRONIC KIDNEY DISEASE	269	173	1.555	1.313	1	1
PSORIASIS	261	11	23.727	25.939	*	1
REFLUX DISORDERS	226	173	1.306	0.780	1	1

Table S15 Ranking of top 10 primary conditions treated during hospitalisation between January 2011 to December 2021, for females with LD.

CONDITION	ADMISSION COUNT	PATIENT COUNT	MEAN ADMISSIONS PER PATIENT	SD OF ADMISSIONS PER PATIENT	MEDIAN ADMISSIONS PER PATIENT	MODE ADMISSIONS PER PATIENT
CANCER	2149	386	5.567	7.836	2	1
CHRONIC KIDNEY DISEASE	742	117	6.342	51.548	1	1
EPILEPSY	631	272	2.320	2.849	1	1
CHRONIC PNEUMONIA	471	177	2.661	1.799	2	2
CHRONIC AIRWAY DISEASES	446	188	2.372	3.008	1	1
CHRONIC ARTHRITIS	386	176	2.193	8.375	1	1
MENTAL ILLNESS	349	179	1.950	1.825	1	1
REFLUX DISORDERS	259	212	1.222	0.626	1	1

Table S15 continued from previous page

CONDITION	ADMISSION COUNT	PATIENT COUNT	MEAN ADMISSIONS PER PATIENT	SD OF ADMISSIONS PER PATIENT	MEDIAN ADMISSIONS PER PATIENT	MODE ADMISSIONS PER PATIENT
DIABETES	203	89	2.281	5.829	1	1
IBD	189	93	2.032	4.228	1	1

Table S16 Common conditions treated during admission of patients between 2011-2021.

	MALE			FEMALE		
	CONDITION	Count	Count %	CONDITION	Count	Count %
1	EPILEPSY	5453	29.410	EPILEPSY	4238	24.097
2	DIABETES	4519	24.373	CHRONIC AIRWAY DISEASES	4002	22.755
3	CHRONIC AIRWAY DISEASES	3619	19.519	DIABETES	3814	21.686
4	MENTAL ILLNESS	3252	17.540	THYROID DISORDERS	2995	17.030
5	CANCER	2243	12.098	MENTAL ILLNESS	2763	15.710
6	CHRONIC KIDNEY DISEASE	1933	10.426	CANCER	2575	14.641
7	CORONARY HEART DISEASE	1783	9.617	CHRONIC KIDNEY DISEASE	2104	11.963
8	THYROID DISORDERS	1660	8.953	CHRONIC ARTHRITIS	1506	8.563
9	CARDIAC ARRHYTHMIAS	1465	7.901	CARDIAC ARRHYTHMIAS	1250	7.108
10	CEREBRAL PALSY	1464	7.896	CEREBRAL PALSY	1118	6.357
11	DEMENTIA	983	5.302	CORONARY HEART DISEASE	1091	6.203
12	CHRONIC ARTHRITIS	970	5.232	DEMENTIA	1019	5.794
13	CHRONIC PNEUMONIA	944	5.091	OSTEOPOROSIS	816	4.640
14	HEART FAILURE	857	4.622	REFLUX DISORDERS	808	4.594
15	REFLUX DISORDERS	851	4.590	HEART FAILURE	648	3.685
16	HEARING LOSS	585	3.155	CHRONIC PNEUMONIA	607	3.451
17	OSTEOPOROSIS	570	3.074	ANAEMIA	571	3.247
18	ANAEMIA	491	2.648	STROKE	394	2.240
19	DYSPHAGIA	467	2.519	HEARING LOSS	379	2.155
20	INSOMNIA	434	2.341	CHRONIC CONSTIPATION	365	2.075
21	VISUAL IMPAIRMENT	428	2.308	IBD	363	2.064
22	STROKE	406	2.190	DYSPHAGIA	343	1.950

Table S16 continued from previous page

	MALE			FEMALE		
	CONDITION	Count	Count %	CONDITION	Count	Count %
23	PSORIASIS	392	2.114	VISUAL IMPAIRMENT	299	1.700
24	CHRONIC CONSTIPATION	336	1.812	CHRONIC PAIN CONDITIONS	273	1.552
25	IBD	318	1.715	INSOMNIA	231	1.313
26	PARKINSONS	278	1.499	MENOPAUSAL AND PRE-MENOPAUSAL	228	1.296
27	PVD	263	1.418	PSORIASIS	227	1.291
28	BRONCHIECTASIS	218	1.176	CHRONIC DIARRHOEA	168	0.955
29	CIRRHOSIS	177	0.955	Peripheral Vascular Disease (PVD)	148	0.842
30	NEUROPATHICPAIN	170	0.917	NEUROPATHICPAIN	145	0.824
31	CHRONIC DIARRHOEA	162	0.874	PARKINSONS	106	0.603
32	BARRETTS OESOPHAGUS	141	0.760	CIRRHOSIS	80	0.455
33	INTERSTITIAL LUNG DISEASE	104	0.561	BARRETTS OESOPHAGUS	77	0.438
34	CHRONIC PAIN CONDITIONS	95	0.512	INTERSTITIAL LUNG DISEASE	75	0.426
35	HYPERTENSION	43	0.232	BRONCHIECTASIS	72	0.409
36	TOURETTE	29	0.156	HYPERTENSION	44	0.250
37	ADDISONS DISEASE	12	0.065	POLYCYSTIC OVARY SYNDROME	37	0.210
38				ADDISONS DISEASE	35	0.199

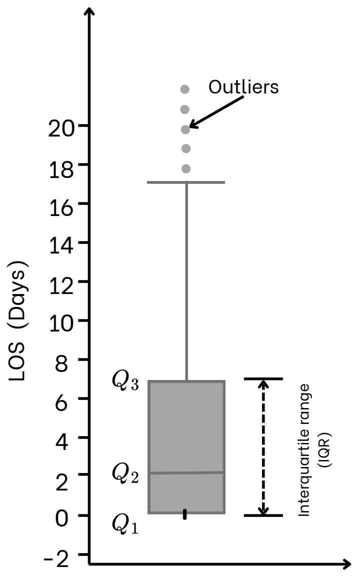

Figure S5. Box plot showing the distribution of LOS across admission records for the combined male and female groups. Outliers in a box plot of all the unique LOS days refer to days that fall significantly outside the range of the majority of the data. Specifically, outliers are defined based on the interquartile range and they help reveal patterns and anomalies that the bulk of the data could obscure.
Remark: The dots indicating 'Outliers' in this figure are illustrations (not real admission points), put in to indicate the presence of admissions above the upper whisker.

Table S17 Top conditions for admissions with LOS ≥ 129 days.

CONDITION	% OCCURRENCE	ADMISSION COUNTS
MENTAL ILLNESS	61	570
EPILEPSY	21.6	202
DIABETES	9.4	88
DEMENTIA	7.3	68
CEREBRAL PALSY	5.1	48
CHRONIC KIDNEY DISEASE	4.4	41
THYROID DISORDERS	4.1	38
CHRONIC AIRWAY DISEASES	4	37
CHRONIC ARTHRITIS	3.2	30
DYSPHAGIA	3.1	29
CARDIAC ARRHYTHMIAS	2.5	23
STROKE	2.4	22

Table S17 continued from previous page

CONDITION	% OCCURRENCE	ADMISSION COUNTS
CHRONIC PNEUMONIA	2.2	21
CORONARY HEART DISEASE	2.2	21
ANAEMIA	2	19
HEART FAILURE	1.9	18
REFLUX DISORDERS	1.9	18
HEARING LOSS	1.6	15
OSTEOPOROSIS	1.5	14
VISUAL IMPAIRMENT	1.3	12
IBD	1.2	11
INSOMNIA	1.1	10
CHRONIC CONSTIPATION	0.9	8
CANCER	0.9	8

Table S18 Chi-square and Binomial tests applied to determine if the categories of each multivariate variable occur with equal probabilities. All variables are described in Table S3.

Hypothesis Test Summary								
	Variable	Test	Male			Female		
			P-value[a]	Test stat	Decision	P-value[a]	Test stat	Decision
1	WIMD	One-Sample Chi-Square Test	0.000	8440.207	Reject hypothesis.	0.000	8175.366	Reject hypothesis.
2	Ethnic group	One-Sample Chi-Square Test	0.000	87412.885	Reject hypothesis.	0.000	84630.100	Reject hypothesis.
3	AUTISM	One-Sample Binomial Test	0.000	2833	Reject hypothesis.	0.000	27962.000	Reject hypothesis.
4	ALCOHOL_HISTORY	One-Sample Chi-Square Test	0.000	21268.570	Reject hypothesis.	0.000	30480.204	Reject hypothesis.
5	SMOKING_HISTORY	One-Sample Chi-Square Test	0.000	19438.225	Reject hypothesis.	0.000	23301.848	Reject hypothesis.

Table S18 continued from previous page

Hypothesis Test Summary								
	Variable	Test	Male			Female		
			P-value[a]	Test stat	Decision	P-value[a]	Test stat	Decision
6	MEDICATIONS	One-Sample Binomial Test	0.000	14492	Reject hypothesis.	0.000	14207.000	Reject hypothesis.
7	PHYSICAL	One-Sample Chi-Square Test	0.000	20305.609	Reject hypothesis.	0.000	20076.616	Reject hypothesis.
8	BMI	One-Sample Chi-Square Test	0.000	115339.196	Reject hypothesis.	0.000	98928.679	Reject hypothesis.
9	AGEGRP_AT_ADMIS_DT	One-Sample Chi-Square Test	0.000	18432.285	Reject hypothesis.	0.000	15325.888	Reject hypothesis.
10	LOSClass	One-Sample Binomial Test	0.000	12063	Reject hypothesis.	0.000	11138.000	Reject hypothesis.

Hypothesis: The categories of each variable occur with equal probabilities.

a. The significance level is .050.

Table S19 Distribution of LOSClass across variable categories.

	MALE		FEMALE	
	Admission Count	LOS>=4 % (Count)	Admission count	LOS>=4 % (Count)
Ethnic group				
Asian	576	0.465 (268)	564	0.355 (200)
Black	75	0.52 (39)	44	0.659 (29)
Other	76	0.342 (26)	56	0.304 (17)
Unknown	6208	0.39 (2421)	5253	0.4 (2101)
White	25340	0.387 (9807)	24051	0.386 (9284)
WIMD				

Table S19 continued from previous page

	MALE		FEMALE	
	Admission Count	LOS>=4 % (Count)	Admission count	LOS>=4 % (Count)
1 (Most Deprived)	9071	0.378 (3429)	8177	0.39 (3189)
2	6947	0.385 (2675)	6855	0.358 (2454)
3	4943	0.42 (2076)	4660	0.423 (1971)
4	4184	0.413 (1728)	4433	0.393 (1742)
5 (Least Deprived)	3068	0.393 (1206)	2811	0.398 (1119)
Unknown	4062	0.354 (1438)	3032	0.382 (1158)
AUTISM				
0	29399	0.392 (11524)	28773	0.391 (11250)
1	2876	0.358 (1030)	1195	0.316 (378)
ALCOHOL_HISTORY				
0	18380	0.406 (7462)	20325	0.377 (7663)
1	3734	0.362 (1352)	1709	0.377 (644)
unknown	10161	0.367 (3729)	7934	0.419 (3324)
SMOKING_HISTORY				
0	16308	0.394 (6425)	17563	0.397 (6973)
1	12809	0.377 (4829)	10325	0.358 (3696)
unknown	3158	0.407 (1285)	2080	0.462 (961)
MEDICATIONS				
0	17298	0.397 (6867)	14482	0.392 (5677)
1	14977	0.38 (5691)	15486	0.384 (5947)
PHYSICAL				
0	14765	0.39 (5758)	13810	0.399 (5510)
1	2541	0.352 (894)	1988	0.293 (582)
unknown	14969	0.394 (5898)	14170	0.391 (5540)
BMI				
normal weight	1084	0.328 (356)	550	0.396 (218)
obesity class I	2869	0.405 (1162)	3901	0.366 (1428)

Table S19 continued from previous page

	MALE		FEMALE	
	Admission Count	LOS>=4 % (Count)	Admission count	LOS>=4 % (Count)
obesity class III	516	0.357 (184)	961	0.344 (331)
pre-obesity	742	0.41 (304)	687	0.378 (260)
underweight	483	0.408 (197)	136	0.449 (61)
unknown	26581	0.389 (10340)	23733	0.393 (9327)
AGEGRP_AT_ADMIS_DT				
0-20	86	0.291 (25)	71	0.366 (26)
20-29	1479	0.372 (550)	1454	0.38 (553)
30-39	4883	0.325 (1587)	4171	0.333 (1389)
40-49	6954	0.356 (2476)	6103	0.326 (1990)
50-59	7677	0.382 (2933)	6840	0.351 (2401)
60-69	6556	0.421 (2760)	6361	0.386 (2455)
70-79	3631	0.446 (1619)	3404	0.541 (1842)
80+	1009	0.588 (593)	1564	0.621 (971)

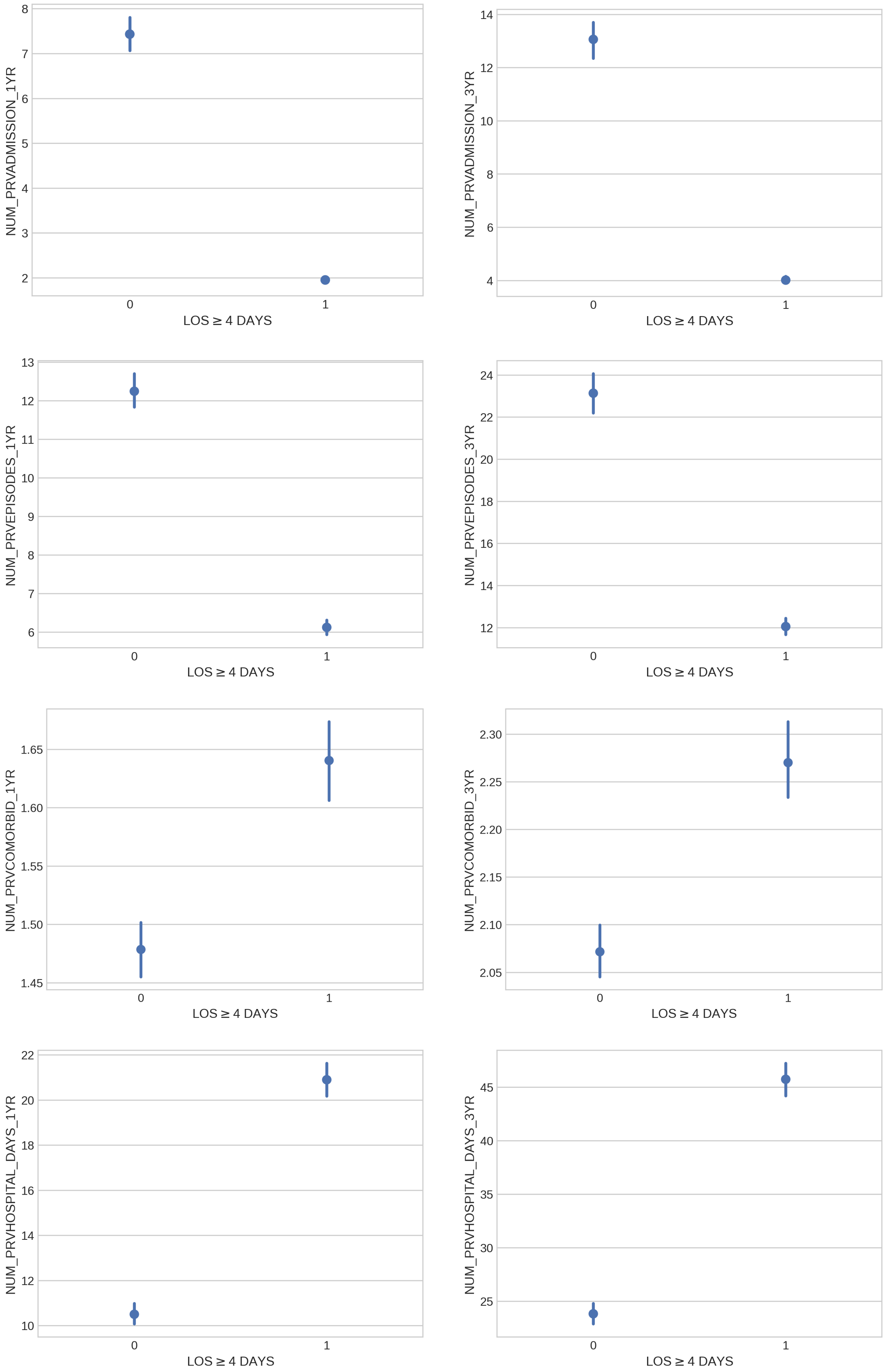

Figure S6. Point plots showing the relationship between the numerical variables and LOS $\geq$ 4 days for males. See Table S3 for description of variables.

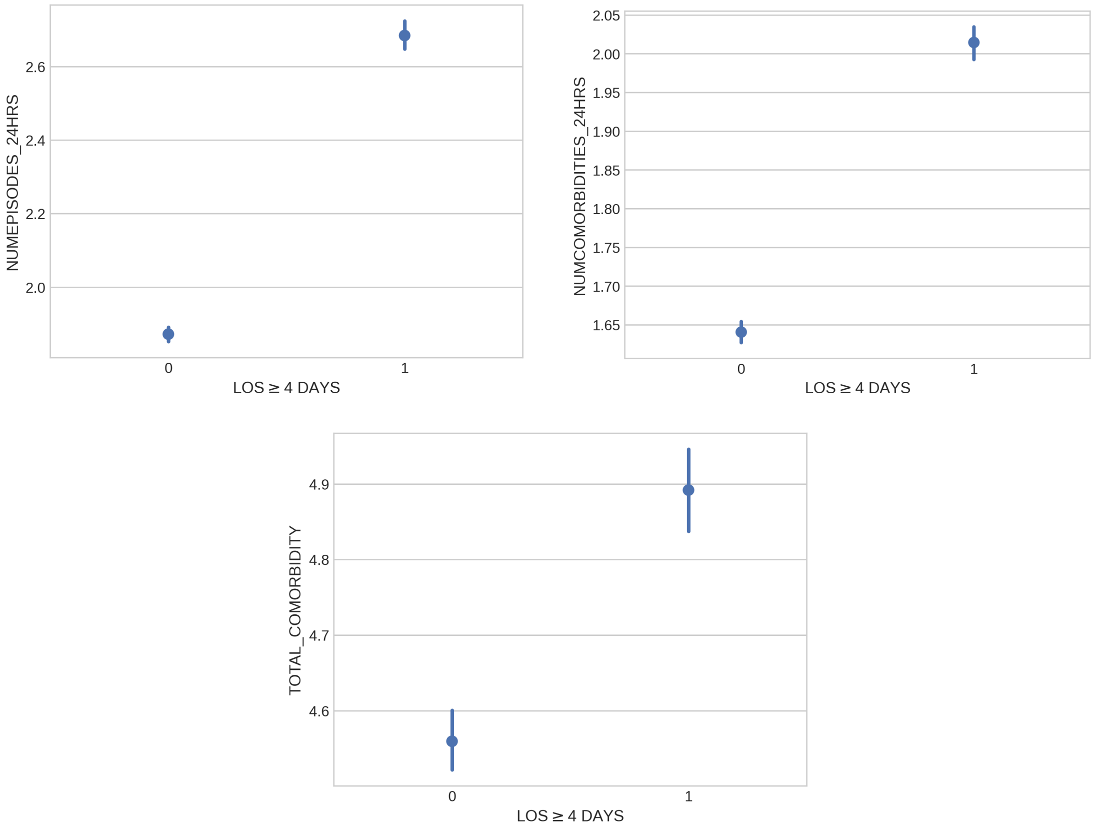

Figure S7. Point plots showing the relationship between the numerical variables and LOS $\geq$ 4 days for males. See Table S3 for description of variables.

Table S20 Comparison of the performance of classifiers used in predicting the LOS for both male and female patients with LD.

	**Male**				**Female**			
	**AUC**	**FNR**	**FPR**	**Balanced Accuracy**	**AUC**	**FNR**	**FPR**	**Balanced Accuracy**
LR	0.742	0.362	0.285	0.677	0.751	0.343	0.292	0.682
RF	0.759	0.224	0.396	0.690	0.756	0.229	0.392	0.689
SVM	0.742	0.420	0.243	0.669	0.747	0.376	0.266	0.679
KNN	0.679	0.375	0.369	0.628	0.681	0.385	0.359	0.628
GBoost	0.742	0.399	0.264	0.668	0.747	0.401	0.251	0.674
HISTGBoost	0.771	0.296	0.303	0.701	0.773	0.278	0.313	0.705
XGBoost	0.763	0.284	0.326	0.695	0.761	0.286	0.331	0.692
NN	0.716	0.496	0.210	0.647	0.723	0.473	0.233	0.647

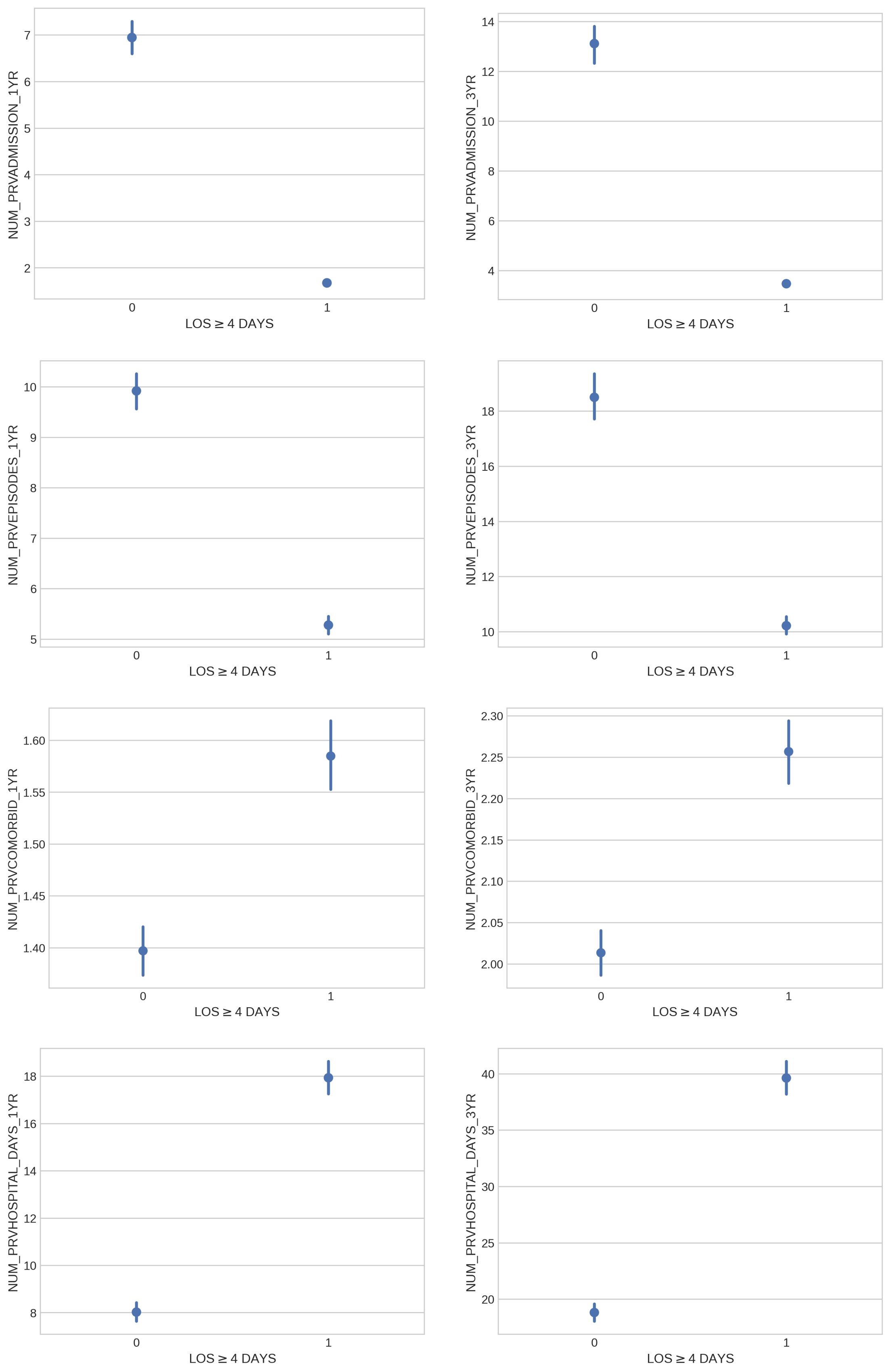

Figure S8. Point plots showing the relationship between the numerical variables and LOS $\geq$ 4 days for females. See Table S3 for description of variables.

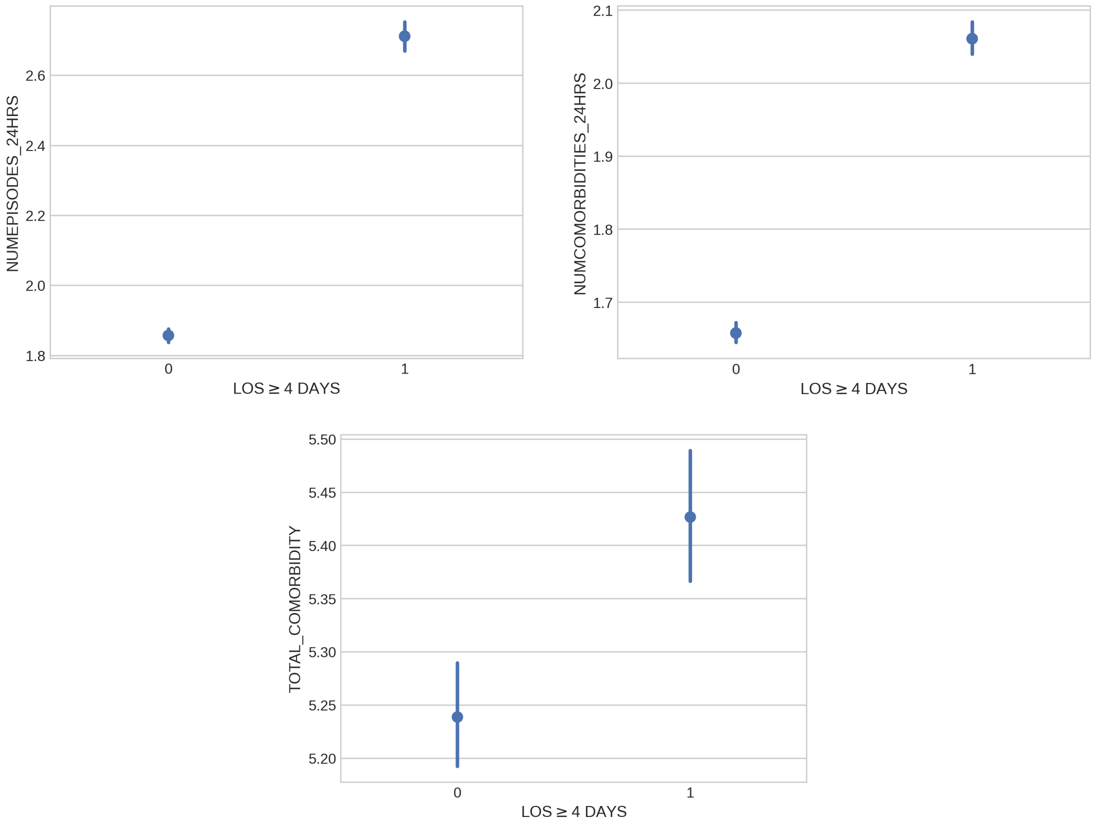

Figure S9. Point plots showing the relationship between the numerical variables and LOS ≥ 4 days for females. See Table S3 for description of variables.

Table S21 Mean performance across the RF classifier after training and testing over 10 random train/test sets.

**MALE**				**FEMALE**			
**AUC (SD)**	**FNR (SD)**	**FPR (SD)**	**Balanced Accuracy (SD)**	**AUC (SD)**	**FNR (SD)**	**FPR (SD)**	**Balanced Accuracy (SD)**
0.758, (± 0.003)	0.226, (±0.005)	0.394, (±0.007)	0.690, (±0.003)	0.762, (±0.003)	0.217, (±0.005)	0.392, (±0.006)	0.696, (±0.002)

Table S22 Performance comparison of the unmitigated RF classifier across Ethnic groups for males. The unmitigated model refers to the classifier without any bias mitigation algorithms applied.

**Ethnic group**	**FNR**	**FPR**	**Balanced Accuracy**
Asian	0.195	0.547	0.629
Black	0.273	0.533	0.597
Other	0.333	0.423	0.622
Unknown	0.234	0.392	0.687

Table S22 continued from previous page

Ethnic group	FNR	FPR	Balanced Accuracy
White	0.222	0.394	0.692

Table S23 Performance comparison of the unmitigated RF classifier across Ethnic groups for females. The unmitigated model refers to the classifier without any bias mitigation algorithms applied.

Ethnic group	FNR	FPR	Balanced Accuracy
Asian	0.216	0.320	0.732
Black	0.167	0.500	0.667
Other	0.111	0.333	0.778
Unknown	0.219	0.398	0.692
White	0.231	0.393	0.688

Table S24 Performance comparison of the unmitigated RF with threshold optimizer and exponentiated gradient across Ethnic groups for males.

	Unmitigated RF Classifier Model		
**Ethnic group**	**FNR**	**FPR**	**Balanced Accuracy**
Asian	0.195	0.547	0.629
Black	0.273	0.533	0.597
Other	0.333	0.423	0.622
Unknown	0.234	0.392	0.687
White	0.222	0.394	0.692
			**ThresholdOptimizer**
**Ethnic group**	**FNR**	**FPR**	**Balanced Accuracy**
Asian	0.297	0.399	0.652
Black	0.318	0.467	0.608
Other	0.333	0.385	0.641
Unknown	0.196	0.438	0.683
White	0.179	0.453	0.684

Table S24 continued from previous page

			Reductions
**Ethnic group**	**FNR**	**FPR**	**Balanced Accuracy**
Asian	0.219	0.581	0.600
Black	0.227	0.733	0.520
Other	0.267	0.346	0.694
Unknown	0.237	0.404	0.679
White	0.228	0.390	0.691

Table S25 Performance comparison of the unmitigated RF with threshold optimizer and exponentiated gradient across Ethnic groups for females.

			Unmitigated RF Classifier Model
**Ethnic group**	**FNR**	**FPR**	**Balanced Accuracy**
Asian	0.216	0.320	0.732
Black	0.167	0.500	0.667
Other	0.111	0.333	0.778
Unknown	0.219	0.398	0.692
White	0.231	0.393	0.688
			**ThresholdOptimizer**
**Ethnic group**	**FNR**	**FPR**	**Balanced Accuracy**
Asian	0.176	0.349	0.737
Black	0.167	0.500	0.667
Other	0.111	0.222	0.833
Unknown	0.191	0.441	0.684
White	0.195	0.437	0.684
			**Reductions**
**Ethnic group**	**FNR**	**FPR**	**Balanced Accuracy**
Asian	0.206	0.314	0.740
Black	0.167	0.500	0.667
Other	0.111	0.278	0.806

Table S25 continued from previous page

Ethnic group	FNR	FPR	Balanced Accuracy
Unknown	0.210	0.413	0.688
White	0.226	0.399	0.687

Table S26 Performance range for the unmitigated RF model and bias-mitigated RF models (threshold optimizer and reductions with exponentiated gradient) for males.

	FNR	FPR	Balanced Accuracy
Unmitigated	0.138	0.156	0.095
Threshold Optimizer	0.155	0.082	0.077
Exponentiated Gradient	0.048	0.387	0.174

Table S27 Performance range for the unmitigated RF model and bias-mitigated RF models (threshold optimizer and reductions with exponentiated gradient) for females.

	FNR	FPR	balanced_accuracy
Unmitigated	0.120	0.180	0.111
Threshold Optimizer	0.084	0.278	0.167
Exponentiated Gradient	0.115	0.222	0.139
